# Data standardization of plant–pollinator interactions

**DOI:** 10.1093/gigascience/giac043

**Published:** 2022-05-26

**Authors:** José A Salim, Antonio M Saraiva, Paula F Zermoglio, Kayna Agostini, Marina Wolowski, Debora P Drucker, Filipi M Soares, Pedro J Bergamo, Isabela G Varassin, Leandro Freitas, Márcia M Maués, Andre R Rech, Allan K Veiga, Andre L Acosta, Andréa C Araujo, Anselmo Nogueira, Betina Blochtein, Breno M Freitas, Bruno C Albertini, Camila Maia-Silva, Carlos E P Nunes, Carmen S S Pires, Charles F dos Santos, Elisa P Queiroz, Etienne A Cartolano, Favízia F de Oliveira, Felipe W Amorim, Francisco E Fontúrbel, Gleycon V da Silva, Hélder Consolaro, Isabel Alves-dos-Santos, Isabel C Machado, Juliana S Silva, Kátia P Aleixo, Luísa G Carvalheiro, Márcia A Rocca, Mardiore Pinheiro, Michael Hrncir, Nathália S Streher, Patricia A Ferreira, Patricia M C de Albuquerque, Pietro K Maruyama, Rafael C Borges, Tereza C Giannini, Vinícius L G Brito

**Affiliations:** Escola Politécnica, Universidade de São Paulo, São Paulo, SP, 05508-010, Brazil; Escola Politécnica, Universidade de São Paulo, São Paulo, SP, 05508-010, Brazil; Departamento de Ecología, Genética y Evolución, Instituto IEGEBA (CONICET-UBA), Facultad de Ciencias Exactas y Naturales, Universidad de Buenos Aires, Buenos Aires, Argentina; Departamento de Ciências da Natureza, Matemática e Educação, Universidade Federal de São Carlos, Rodovia Anhanguera km 174, Araras, São Paulo, Caixa Postal 153. CEP 13600-970, Brazil; Instituto de Ciências da Natureza, Universidade Federal de Alfenas, Rua Gabriel Monteiro da Silva 700, Alfenas, Minas Gerais, 37130-001, Brazil; Embrapa Agricultura Digital, Empresa Brasileira de Pesquisa Agropecuária (Embrapa), Campinas, SP, Brazil; Escola Politécnica, Universidade de São Paulo, São Paulo, SP, 05508-010, Brazil; Jardim Botânico do Rio de Janeiro, R. Pacheco Leão 915, Rio de Janeiro, Rio de Janeiro, 22460-030, Brazil; Departamento de Botânica, Universidade Federal do Paraná, Curitiba, Paraná, Brazil; Jardim Botânico do Rio de Janeiro, R. Pacheco Leão 915, Rio de Janeiro, Rio de Janeiro, 22460-030, Brazil; Laboratório de Entomologia, Embrapa Amazônia Oriental, Trav. Dr. Enéas Pinheiro, s/n°, Bairro do Marco, Belém, Pará, 66095-903, Brazil; Faculdade Interdisciplinar de Humanidades, Centro Multiusuário de Pesquisa em Ciência Florestal (MULTIFLOR), Universidade Federal dos Vales do Jequitinhonha e Mucuri, Diamantina, Minas Gerais, 39100-000, Brazil; Escola Politécnica, Universidade de São Paulo, São Paulo, SP, 05508-010, Brazil; Instituto Tecnológico Vale. Rua Boaventura da Silva, 955, 66055-900, Belém, Pará, Brazil; Instituto de Biociências, Universidade Federal de Mato Grosso do Sul, Campo Grande, Mato Grosso do Sul, Brazil; Laboratório de Interações Plant-Animal (LIPA), Centro de Ciências Naturais e Humanas (CCNH), Universidade Federal do ABC, Alameda da Universidade, s/nº, Anchieta, São Bernardo do Campo, São Paulo, Brazil; Escola de Ciências da Saúde e da Vida, Pontifícia Universidade Católica do Rio Grande do Sul, Porto Alegre, RS, 90619-900, Brazil; Departamento de Zootecnia, Campus Universitário do Pici, Universidade Federal do Ceará, Centro de Ciências Agrárias, Fortaleza, CE, Brazil; Escola Politécnica, Universidade de São Paulo, São Paulo, SP, 05508-010, Brazil; Departamento de Biociências, Universidade Federal Rural do Semi-Árido, Av. Francisco Mota, n° 572, Presidente Costa e Silva, Mossoró, RN, 59625-900, Brazil; Department of Biological and Environmental Sciences, Cottrell Building, University of Stirling, Stirling FK9 4LA, Scotland, United Kingdom; Embrapa Recursos Genéticos e Biotecnologia, Brasília, Distrito Federal, Brazil; Escola de Ciências da Saúde e da Vida, Pontifícia Universidade Católica do Rio Grande do Sul, Porto Alegre, RS, 90619-900, Brazil; Departamento de Ecologia, Instituto de Biociências, Universidade de São Paulo, São Paulo, Brazil; Escola Politécnica, Universidade de São Paulo, São Paulo, SP, 05508-010, Brazil; Laboratório de Bionomia, Biogeografia e Sistemática de Insetos (BIOSIS), Instituto de Biologia (IBIO), Universidade Federal da Bahia, 40170-115 Salvador, Bahia, Brazil; Laboratório de Ecologia da Polinização e Interações (LEPI), Programa de Pós-graduação em Botânica, Programa de Pós-graduação em Zoologia, Instituto de Biociências, Universidade Estadual Paulista, Botucatu, SP, Brazil; Instituto de Biología, Facultad de Ciencias, Pontificia Universidad Católica de Valparaíso, Valparaíso, Chile; Programa de Pós-Graduação em Ecologia / INPA-V8 - Instituto Nacional de Pesquisas da Amazônia, Av. André Araújo 2936, Petrópolis, 69067-375, Manaus - AM, Brazil; Instituto de Biotecnologia, Universidade Federal de Catalão, Catalão, Goiás, Brazil; Departamento de Ecologia, Instituto de Biociências, Universidade de São Paulo, São Paulo, Brazil; Programa de Pós-Graduação em Biologia Vegetal, Departamento de Botânica, Universidade Federal de Pernambuco, Recife, PE 50670-901, Brazil; Instituto Federal de Educação Ciência e Tecnologia de Mato Grosso, Avenida Sen. Filinto Müller, 953 - CEP: 78043-400 - Cuiabá, MT, Brazil; Associação Brasileira de Estudos das Abelhas (A.B.E.L.H.A.), São Paulo, SP, 04535-001, Brazil; Departamento de Ecologia, Universidade Federal de Goiás, Campus Samambaia, Goiânia, Brazil Centre for Ecology, Evolution and Environmental Changes (cE3c), University of Lisboa, Lisbon, Portugal; Departamento de Ecologia, Centro de Ciências Biológicas e da Saúde, Universidade Federal de Sergipe, Avenida Marechal Rondon s/n, São Cristóvão, Sergipe, 49100-000, Brazil; Universidade Federal da Fronteira Sul, R. Major Antônio Cardoso 590, Cerro Largo, Rio Grande do Sul, 97900-000, Brazil; Departamento de Fisiologia, Instituto de Biociências, Universidade de São Paulo, Rua do Matão, 321, Travessa 14, São Paulo, São Paulo, 05508-900, Brazil; Department of Biological Sciences, University of Pittsburgh, Pittsburgh, PA,15260, United States of America; Environmental Sciences Department, Federal University of São Carlos, São Paulo, Brazil; Federal University of Maranhão, Dept. Biology, Dom Delgado University Town, Bacanga, São Luís, MA, Brazil; Centro de Síntese Ecológica e Conservação, Departamento de Genética, Ecologia e Evolução, Instituto de Ciências Biológicas, Universidade Federal de Minas Gerais, Belo Horizonte, Minas Gerais, Brazil; Instituto Tecnológico Vale. Rua Boaventura da Silva, 955, 66055-900, Belém, Pará, Brazil; Instituto Tecnológico Vale. Rua Boaventura da Silva, 955, 66055-900, Belém, Pará, Brazil; Instituto de Biologia, Universidade Federal de Uberlândia, Rua Ceará sn, Uberlândia, Minas Gerais, 38.405-302, Brazil

**Keywords:** biodiversity information, Darwin Core, vocabulary of terms, pollination, pollinator, biodiversity informatics

## Abstract

**Background:**

Animal pollination is an important ecosystem function and service, ensuring both the integrity of natural systems and human well-being. Although many knowledge shortfalls remain, some high-quality data sets on biological interactions are now available. The development and adoption of standards for biodiversity data and metadata has promoted great advances in biological data sharing and aggregation, supporting large-scale studies and science-based public policies. However, these standards are currently not suitable to fully support interaction data sharing.

**Results:**

Here we present a vocabulary of terms and a data model for sharing plant–pollinator interactions data based on the Darwin Core standard. The vocabulary introduces 48 new terms targeting several aspects of plant–pollinator interactions and can be used to capture information from different approaches and scales. Additionally, we provide solutions for data serialization using RDF, XML, and DwC-Archives and recommendations of existing controlled vocabularies for some of the terms. Our contribution supports open access to standardized data on plant–pollinator interactions.

**Conclusions:**

The adoption of the vocabulary would facilitate data sharing to support studies ranging from the spatial and temporal distribution of interactions to the taxonomic, phenological, functional, and phylogenetic aspects of plant–pollinator interactions. We expect to fill data and knowledge gaps, thus further enabling scientific research on the ecology and evolution of plant–pollinator communities, biodiversity conservation, ecosystem services, and the development of public policies. The proposed data model is flexible and can be adapted for sharing other types of interactions data by developing discipline-specific vocabularies of terms.

## Background

### Introduction

Pollination is a key natural process that provides indispensable ecosystem services and safeguards agricultural production and food security worldwide [1]. Almost 90% of flowering plant species [2], including more than half of the global crop species [3], rely to some degree on animal pollination for their reproduction [4, 5]. Concerned with the current global biodiversity crisis and its impacts on ecosystems and human health, the Convention on Biological Diversity [6] and the Intergovernmental Science–Policy Platform on Biodiversity and Ecosystem Services (IPBES) [7] acknowledged the importance of plant–pollinator interactions for ecosystem functioning and sustainable agriculture [8]. Although large data sets of plant–pollinator interactions data have become available worldwide, great challenges remain regarding data storage and standardization. These issues need to be solved to enable the development of integrative studies that allow attaining broad-scale knowledge on species biology, phenology, and evolution, as well as to support the decision-making process for pollinator conservation. Before IPBES, many initiatives and funding programs were created to promote and support research and conservation of pollinators and plant–pollinator interactions adopting the concept of open data. Among the most prominent are the International Pollinators Initiative–The São Paulo Declaration on Pollinators [9], the Global Action on Pollination Services for Sustainable Agriculture of the Food and Agriculture Organization of the United Nations (FAO, [10]), the United States Geological Survey (USGS) Pollinators Conservation Program [11], and the European Union Pollinators Initiative [12]. Nevertheless, many data gaps still exist regarding plant–pollinator interactions (see, e.g., Wolowski et al. [13] for an analysis of native species in the Atlantic forest). Initiatives like the IPBES have demanded quick access to high-quality spatial and temporal data of species occurrences, their interspecific relations, and the environmental effects on biotic interactions. These high-quality data have the potential to improve our knowledge about ecological and evolutionary processes guided by interspecific interactions, as well as to assist in planning and decision-making for biodiversity conservation and restoration [14].

Primary data on pollinators are becoming increasingly available online and can be accessed from a large number of data repositories. Moreover, many initiatives have also been created to facilitate and to stimulate the dissemination of pollinators and plant–pollinator interactions data, such as the InterAmerican Biodiversity Information Network–Pollinators Thematic Network (IABIN-PTN), the WebBee [15], the UK plant–pollinator interactions database [16], and the Plant–Pollinator Interaction Explorer [17]. There are also more general initiatives that aim to organize data of all types of biotic interactions, for example, the projects Global Biotic Interactions–GloBI [18], Gulf of Mexico Species Interaction–GoMexSI [19], Mangal [20], Interaction Web DataBase (IWDB) [21], Kelpforest Database [22], the LifeWebs project [23], the GlobalWeb [24], and the Web of Life [25]. Despite the increasing availability, there remain serious data gaps and biases. For instance, there is a larger amount of interaction data from temperate and high-latitude regions compared to the tropics [26, 27], hampering the assessment of global patterns such as latitudinal gradients [28, 29]. Species interaction data, especially binary matrices or binary networks (i.e., presence/absence of interaction), can also be found in many scientific papers, but detailed information on each interaction and species traits is still sparse in the literature. Having scattered information has hindered answering urgent questions about the roles of species and their interactions within communities and ecosystems, and their impact on ecosystem functions and services [30–32], as well as understanding how pollinators behave or with whom they interact in different types of ecosystems or biomes.

Most currently available species interaction data sets do not adopt any standard for data or metadata capture and annotation (e.g., Allen-Perkins et al. [33]). Moreover, for those that do, the lack of appropriate data standards largely contributes to the dispersion and heterogeneity in the data. Thus, data aggregation relies on laborious and repeated transformations of the original data sets into custom, nonstandardized formats, making data integration and discovery a costly and time-consuming process. In addition, data on interactions recorded by different studies may impose limitations to the generalization of conclusions due to variation in sampling methods and research objectives, usually not documented in the metadata (e.g., Pimm et al. [34], Beas-Luna et al. [22]). As a result, species interaction data are often insufficient or biased for many types of analyses.

Broad-scale analyses require data to satisfy the FAIR principles (i.e., Findable, Accessible, Interoperable, and Reusable data; Wilkinson et al. [35]). Fulfilling such criteria is challenging, and it is essential that biodiversity standards (e.g., Findable, Accessible, Interoperable, and Reusable data) aid in meeting those principles, as they enable comparison of data from different contexts, shared through different open-access global databases.

Findable, Accessible, Interoperable, and Reusable data (hereafter DWC; Wieczorek et al. [36]) is a standard for sharing data about life on Earth as documented by observations, specimens, samples, and related information. DWC was ratified as a standard in October 2009 by the Biodiversity Information Standards (TDWG) organization. Since then, it has been adopted by several communities around the globe. The most prominent case of DWC adoption is perhaps the Global Biodiversity Information Facility [37], which aggregates more than 1.9 billion biodiversity data records, as of January 2022. The DWC standard and other standards for biodiversity data and metadata, such as Access to Biological Collection Data (ABCD [38]), Audubon Core [39], and Ecological Metadata Language (EML; Jones et al. [40]), constitute a great advance in biological data sharing and aggregation, supporting the development of studies and science-based decision-making. However, a general, adaptive, and comprehensive solution for biological interaction data standardization, including plant–pollinator interactions, is still not available.

Biological interactions usually include data that cannot be adequately represented by the DWC standard as it currently stands, because it lacks appropriate terms to document them in detail. Also lacking is a common model to express important components of the phenomena, such as the type, direction, effects, and outcomes of an interaction. However, DWC is flexible enough to be extended and new terms and controlled vocabularies may be created to accommodate new use cases. For example, DWC has been extended to support standardization of genomic data [41, 42] and zooarchaeological data [43], and several other extensions are currently being used or are in development by the community [44]. The latest version of DWC (version 2021-07-15) also incorporates 4 controlled vocabularies of values.

Every (pairwise) interaction involves 2 organisms or 2 groups of taxonomically homogeneous organisms that perform a coaction at a particular place and time [45]. While the taxonomic, spatial, and temporal information about the occurrences of such organisms or group of organisms can be documented using DWC, there is no formal or recommended process to express the association of such occurrences and the particularities of an interaction.

Despite that, biological interactions data have been documented using many different approaches, including the adoption of the “Association terms” from the DWC standard (i.e., dwc:associatedTaxa and dwc:associatedOccurrences) and the dwc:ResourceRelationship class. We also find some nonconventional ways to document interactions using the terms dwc:occurrenceRemarks and dwc:dynamicProperties and those in the class dwc:MeasurementOrFact (MoF). There is also a non-standard DWC “association extension” [46] developed by Encyclopedia of Life (EOL) that focuses primarily on taxonomic characteristics of the interactions, instead of their ecological and functional aspects.

To extend our capacity to share interactions data, in this article, we present a vocabulary of terms to document plant–pollinator interactions developed by a community of specialists and a data model to use the vocabulary based on DWC. The remainder of the article is organized as follows: first, we provide an overview of the previous initiatives regarding plant–pollinator data, which have paved the way for this work. We then introduce and discuss the process of community-driven vocabulary development and present the plant–pollinator interactions vocabulary itself. Last, we present the use of DWC to document biological interactions, including the plant–pollinator interaction data model for representations using DwC-Archives, XML, and RDF, and draw some conclusions.

#### Historical overview on plant–pollinator interactions data standardization

The plant–pollinator interactions vocabulary presented here started to be assembled in 2006, based on the demand from the IABIN-PTN. The initiative aimed at digitizing pollinator data for the Americas, including information on species occurrences, usually provided by biological collections and museums, as well as ecologically relevant information on plant–pollinator interactions that were at the time seldom digitized [47]. In a joint effort with the FAO, a first solution proposed the use of 3 extensions to the existing Findable, Accessible, Interoperable, and Reusable data (named DWC v1.4, not yet a TDWG standard). They consisted of (i) a generic Interaction Extension intended to represent any observed interaction between 2 individuals, not restricted to pollinators or plants; (ii) a Pollination Extension, including additional data specific to plant–pollinator interactions, for instance, pollen or nectar removal; and (iii) an Environmental Measurement Extension to include the environmental conditions during the observation or collecting event. That proposal was published on the Darwin Core wiki [48] for broader discussion within the TDWG community and attracted some attention. Despite the benefits of being a more generic approach, the discussions showed that it would require a lot of effort to reach consensus, and given the time constraints of the project, it was decided to focus only on plant–pollinator interactions. The subsequent version of the so-called Interaction Schema treated each interaction record as a triad: one DWC record for each of the 2 interacting organisms and an interaction record that referenced the individual DWC records by means of their globally unique identifiers. The interaction record also included data about the type of interaction (e.g., collecting pollen, collecting nectar), observer, location, and date/time of the interaction. This approach allowed multiple interaction records to be associated with the same interacting individual [49].

Further simplification led to adopting only 2 terms to characterize the interaction: one field for the type of interaction and another field for remarks, typically used for a bibliographic reference of the interaction. That was, finally, the solution adopted for the system and tools developed during the IABIN-PTN project [50]. The same approach was used for digitization of interaction data collected within a joint initiative from United Nations Environment Programme (UNEP) and the Global Environment Facility (GEF)/FAO Global Pollination Project on the “Conservation and Management of Pollinators for Sustainable Agriculture, through an Ecosystem Approach,” which involved partners in 7 countries: Brazil, Ghana, India, Kenya, Nepal, Pakistan, and South Africa [51]. Overall, those projects have enabled the digitization of thousands of plant–pollinator records that follow the same template and can, thus, be reused more easily.

Knowing the potential and importance of adding richer data content to each interaction, another attempt was made to further evolve the pollinator interaction data standard. Also supported by FAO, a survey of potential descriptors of plant–pollinator interactions was conducted with researchers from 5 continents [51]. The 23 participants shared the data fields they used to digitize interaction data for their research purposes. As their research questions varied, so did the fields they used in their spreadsheets (most cases) or databases (a few cases). The result of that compilation was a very long list of data fields (more than 200), which included terms related to the plant (taxonomy, traits), the potential pollinator or flower visitor (taxonomy, traits), the experimental setting and protocol, the environment, the outcome of the interaction (such as fruit set), and references, among others. As for the interaction-related fields, some referred to pollinators' behavior and some to resources collected, interaction frequency, results, or outcomes. Many of the descriptors suggested by different authors seemed to be synonyms (as one might expect), but they were not accompanied by a clear description of their meaning and form of usage (semantics and syntax), which made it difficult to compare and coalesce them. That list was clearly very valuable but needed a detailed evaluation and intense work to compare, categorize, sort, and define the fields so as to identify a good set of candidate terms. That required experts in the fields involved—pollination ecology, botany, zoology, information, and computing science—to create a community-driven vocabulary, which is critical for the development of a proposal that really reflects the vision and the needs of a broad community, a prerequisite for a data standard [52], and fosters its subsequent adoption.

With its founding members aware of the previous context, the Brazilian Network of Plant–Pollinator Interactions (REBIPP) was established in 2016 with the aim of encouraging scientific, educational, and outreach activities related to pollination biology. REBIPP is a collaborative network of specialists in pollination biology, researchers of plant–pollinator interactions in its various scales and dimensions, and one of its original objectives was the development of a Brazilian Plant–Pollinator Interaction Database. Having to deal with the current standards shortcomings firsthand and increasingly involved, members of the network were keen to take a step further into developing a more comprehensive solution. Building on the momentum of this community engagement, REBIPP seized the opportunity to broaden its original objectives, welcoming other members of the international community, to jointly develop a solution for sharing standardized plant–pollinator interactions data.

## Community-driven vocabulary development

During 2017 and 2018, specialists on pollination biology and information science from the Brazilian and Chilean networks on plant–pollinator interactions met in 4 workshops to review and discuss those descriptors of plant–pollinator interactions defined in the previous initiatives mentioned above. The meetings aimed to engage all participants in discussions and to reach consensus about the terms that would compose the standardized vocabulary for plant–pollinator interaction data. Specialists worked simultaneously in 3 task groups (plant, animal, and interaction groups), each focused on the revision and definition of specific terms. The first 2 groups focused on terms to describe relevant plant or animal traits, while the interaction task group reviewed the descriptors that characterize the interactions. In order to facilitate reconciling the vocabularies from different groups, a common template was used (Table 1). Periodically, the task groups engaged in all-hands discussions, so that each group could get acquainted with the progress and decisions made by the others.

**Table 1:** Template used to define the terms in the plant–pollinator interactions vocabulary

Term Label: Flower Opening Type	
**Identifier**	http://rs.rebipp.org.br/ppi/terms/flowerOpeningType
**Class**	Flower
**Definition**	The type of flower describing whether the flower’s corolla opens or not, exposing its reproductive parts
**Comments**	Recommended best practice is to use a controlled vocabulary.
**Details**	Proctor, M. P. et al. 1996. The natural history of pollination. HarperCollins. Inouye DW, Favre DW, Lanum JA,
	Levine DM, Meyers JB, Roberts MS, Tsao FC, Wang Y-Y. 1980. The effects of nonsugar nectar constituents on
	estimates of nectar energy content. Ecology 61: 992–996
**Protocol**	Observation of the floral development from the bud stage to senescence (Dafni et al. 2005).
**Controlled Vocabulary**	cleistogamous; chasmogamous; both
**Examples**	cleistogamous; chasmogamous; both

*Term Label*: a human readable name; *Identifier*: a unique Internationalized Resource Identifier (IRI) for the term in namespace; *Class*: the category in which a term is defined. It is not a formal class definition (aka rdf:Class); *Definition*: term definition in a human readable form; *Comments*: any additional comments to the term definition and its use; *Details*: a reference for the concept represented by a term; *Protocol*: recommended protocols to measure or to record the value for the term (if applicable); *Controlled Vocabulary*: list of recommended values, such as terms from existing thesauri or ontologies (if applicable).

Reaching a consensus on terms and their definitions among members of each group was challenging, since researchers have different views and concerns about which data are important and must be represented in a standard, as expected in any scientific field (e.g., Tremblay et al. [53]). Thus, we ended up having a list of 278 terms, many of which were useful only in very specialized research protocols and experiments, rarely collected, or measured. Following the metadata principle of simplicity [54–56], on the premise that a standard with too many terms is difficult to use and complexity imposes barriers to its adoption, we started a second round of terms review. For this, the task groups worked together on the whole set of terms to refine the definitions and reduce the list to a core of important concepts related to plant–pollinator interactions. With a reduced list of 278 terms, we built a draft version of the vocabulary, and specialists in biodiversity, informatics, and information science worked together to validate and refine it, ensuring it would be compliant with current standard wording and practices. Finally, we performed a “Community Review” among all participant members to solve any conceptual and practical problems and validate the vocabulary using real data examples. To conduct the review, we used GitHub Issues Tracking [57], which was essential for the process to be transparent and open access, and the template to formalize the definitions of terms, which also helped the organization of the vocabulary.

After almost 3 years of collaborative and voluntary work, the first version of the plant–pollinator standard was concluded. The vocabulary includes 48 new terms specifically defined for plant–pollinator interactions (see Supplementary Material), which can be accessed through the open access and stable repository [58]. Additionally, we provide controlled vocabularies for many terms that bring the definition of new controlled vocabulary (CV) terms or import terms from other existing vocabularies [59]. It is important to note that the terms discarded from this first release of the vocabulary and their history have been kept in the GitHub repository and can be revisited in the future as the vocabulary evolves.

### Guidelines to collaborative creation of a new vocabulary of terms

The collaborative creation of a vocabulary involves many challenges with different levels of complexity. This complexity is partially related to the empirical and sociological components of a collaborative and democratic community. In order to facilitate and help other biodiversity information communities in the creation of their own standards and vocabularies, we elaborated a set of guidelines covering aspects from the conceptualization to the adoption of a vocabulary. The guidelines were elaborated mainly based on our experience during the creation of the plant–pollinator interaction vocabulary. Although the technical details of how to build a standard are already documented in the TDWG Vocabulary Maintenance Standard (VMS Group [60]) and in the TDWG Standards Documentation Standard (SDS Group [61]), there is no guidance on how a community should organize itself and how members should collaborate to democratically reach an agreement. For that reason, here we propose a workflow that can be used and adjusted by other communities according to their needs and requirements.

The workflow is summarized in the following steps:

- **Identification of key stakeholders:** this is probably the most critical step. Engagement from the community is vital not only for the development of a vocabulary but, more important, for its later adoption. It is important for the members of the community to clearly understand the benefits of data standardization and also to give them a sense of ownership over the resulting products. However, there should be a careful balance between representativeness and group size. The formation of smaller and more homogeneous working groups focusing on specific parts of the vocabulary being created should follow some predefined criteria. This promotes having more operative and efficient groups. In our case, the community was divided in 3 groups according to each member’s expertise in botany, zoology, and ecology. The size of the groups is dependent on the size of the whole community, but working groups that are unbalanced in size may lead to biased perceptions and definitions of the main topic.
- **A formal definition of the main topic:** the main topic (including main goals and challenges) must be defined early in the creation of the vocabulary. Conceptualizing the main topic may involve the abstraction of complex and sometimes ambiguous concepts (in the case of pollination ecology, e.g., “species,” “specimen,” “traits,” “functional traits,” “legitimate pollinator,” “floral visitor”), and thus it is essential to have a robust and formal definition of the subject matter. Expert knowledge may facilitate the definition of the main topic, but it should not be the only source of knowledge (e.g., literature, glossaries, nomenclature codes should also aid the process). The formal definition needs to be based on an agreement reached by the community to set a clear scope and avoid ambiguities and conflicting concepts. The definition may be created by borrowing concepts and terms from other standards and vocabularies.
- **Collaborative compilation of an initial set of terms:** to describe the data domain (what we refer to as “variables” or “descriptors” to avoid confusion with a formal term that will be part of the standard or the vocabulary). This may include assessment of terms from existing standards. When reusing terms from existing standards and vocabularies, attention should be given to avoid misunderstanding of the concepts represented by the terms. For that reason, this step should only focus on the description of the concepts (“descriptors”) instead of their representation as a formal vocabulary term (i.e., an entity representing a concept) [60]. The working groups should collaborate to compile an inclusive set of descriptors that contemplates a general understanding of the concepts, but conflicting concepts may be accepted depending on the heterogeneity of the whole community. In more homogeneous communities where members already have a consensus about the concepts to be represented by the vocabulary, the following steps and the creation of the vocabulary may be simplified.
- **Review and refinement:** the initial set of descriptors provides a preliminary definition for the terms. Those terms should be subjected to rounds of review to refine their definitions and examples of usage and to elaborate glossaries for controlled vocabularies (if applicable). This is the step in which terms can be merged if their definitions reflect the same concept or split into 2 or more terms if a need is identified. If the set of terms includes a large number of conflicts, it can be split into multiple more specific sets (which may lead to multiple vocabularies). Alternatively, the community may try to deal with conflicting terms by defining more general concepts. Terms can also be excluded. We do not recommend adding new descriptors at this step (unless by splitting existing descriptors or by adopting terms from existing vocabularies) because the addition of new terms would result in unnecessary vocabulary growth and a larger number of review rounds to reach a consensus. The working groups should focus on the review and refinement of the descriptors related to their objectives and use cases, but they may collaborate with each other, especially to adopt terms from existing data standards. New terms should only be defined if they are not already defined by other data standards. The process described in this step should be iterated until full consensus is reached.
- **Minting new terms:** the refined set of terms should be mapped to other community vocabularies (e.g., DWC), as should the recommended controlled vocabularies (if applicable). Sometimes a term can be similar to a term already defined in another available vocabulary but with slightly different semantics or formatting. Whenever possible, it is recommended to review the new term to match an existing term definition. If this is not feasible or appropriate, the new term should be created and added to the draft version of the vocabulary. It is also possible to propose a change to an existing term in other standards.
- **Elaborate a representation model:** it is also advisable to elaborate at least 1 representation model for the vocabulary and document its usage under different schemas. Our recommendation is to at least declare the terms using RDF to improve the interoperability with other communities but also to provide other schemas if possible, such as Darwin Core Archives and XML. It is important to consider solutions already adopted by a broader community in order to maintain the interoperability and consistency among models and schemas. If the data model is too complex or the vocabulary cannot be represented in one of the chosen schemas, go back to step 4 and try to refine the terms in order to reduce any dependencies among the terms (e.g., a term for which the interpretation depends on the value of another term, a multilevel of many-to-one or many-to-many relationships between the terms).
- **Validation:** compiling a set of real data covering different scientific questions and verifying if the vocabulary can capture all or the most relevant information needed for each use case. If any issue is detected (e.g., missing or conflicting definitions) or there is any ambiguity in the definition of the terms, go back to step 4 and refine the set of terms to overcome it.
- **Make the standard broadly available:** (e.g., through formal publication or collaborative platforms) so that members of other communities can openly access it for use and be involved in its evolution. For this purpose, GitHub [62] has proven to be a useful platform for tracking and maintaining standards. However, keep in mind that other layouts may be needed for those audiences that are not familiar or comfortable with GitHub repositories. Creating a simple, friendly webpage with a description of the vocabulary and its purpose and where users can browse for terms and definitions may be considered (a model for this is the DWC Quick Reference Guide [6].

### Data quality and controlled vocabularies

The adoption of controlled vocabularies contributes significantly to data quality and interoperability of biodiversity data sets from the same and other communities, as it makes data easier to find and use [64]. Although the plant–pollinator interactions vocabulary does not restrict how data are captured under each term, it provides some guidelines for the adoption of thesauri and ontologies, when available. There are many ontologies available that can enrich data description and annotation and that are relevant to plant–pollinator interactions data, such as the Plant and the Plant Trait Ontologies [65], the Phenotype and Trait Ontology [66], the Flora Phenotype Ontology [67], the Hymenoptera Anatomy Ontology [68], the Vertebrate Ontology [69], and Uberon [70]. Moreover, ontologies are also available for environment description, like the Environment Ontology [71], and biotic interactions, such as the Relations Ontology [72]. Recommendations to use particular controlled vocabularies are not meant to be normative, and since there are values that cannot be mapped to any existing controlled vocabularies, we provide some for specific terms. As new or revised vocabularies emerge from the community, such recommendations should be easily amended, accompanying and facilitating the evolution of the vocabulary.

## A plant–pollinator interactions vocabulary

### Defining an interaction

The potential uses of the broad and heterogeneous term *interaction data* need to be circumscribed by providing a common definition and understanding shared by members of different communities. Achieving an agreement in the definition of an “interaction” in its broadest context (e.g., “behavioral interaction,” “ecological interaction”) proved to be challenging, as expected, provided the long-standing debate around the subject in the ecological literature [73]. Even when restricted to plant–pollinator interactions, the diversity of background knowledge and expertise in a multidisciplinary field such as pollination biology resulted in divergent and conflicting concepts of what an “interaction” is according to different perspectives (e.g., plant-centered perspective, animal-centered perspective, interaction among species, interaction among individuals).

Those conflicting concepts led to an initial proposition of a great number of variables to describe and characterize an “interaction.” In addition, the high level of abstraction of these concepts requires a subjective causal inference that is beyond what primary data can represent. Unlike recording the occurrence of an organism, which is restricted to spatial and temporal scales, recording an “interaction” also involves a subjective human interpretation about the biological meaning and effects of such interaction. There are also several contingencies (e.g., for competitive exclusion; Pedruski et al. [74]) and multiple definitions being adopted (e.g., for mutualism, Mazancourt et al. [75]; and symbiosis, Martin and Schwab [76]). Despite discrepancies, there is a consensus in the community that an “interaction” is a context-dependent action that occurs at a particular location during some time. By “context dependent,” we mean that interactions are dependent on the habitat and the species- or individual-level traits of the interacting organisms [77], as well as on the presence/absence of individuals of other species (e.g., higher-order interaction; Werner [78]).

Another widely discussed topic is related to the level at which interactions are recorded. While some members of the community emphasize that an interaction should be documented at the species level, others argue that it should be documented at the individual level [79]. Species-level interactions have been historically documented, especially in community ecology, yet they consist of a summary of interactions that are actually recorded in the field at the individual level [80]. Since DWC is “primarily based on taxa, and their occurrence in nature as documented by observations, specimens, samples, and related information,” it makes sense to document interactions at the individual level. Additionally, species-level interactions could be derived from aggregating individual-level interactions but not in the opposite direction. Thus, we adopted the following definition of an interaction:

**Interaction:** a context-dependent action that a particular organism or group of organisms (considered to be taxonomically homogeneous) performs on another particular organism or group of organisms (taxonomically homogeneous) living together in a community at a particular location during some time.

An organism is defined as “a material entity that is an individual living system, such as animal, plant, bacteria or virus, that is capable of replicating or reproducing, growth and maintenance in the right environment. An organism may be unicellular or made up, like humans, of many billions of cells divided into specialized tissues and organs” [81].

Our definition emphasizes the individual perspective of the interactions in contrast to the species perspective [80]. Additionally to the spatial and temporal elements of an interaction, there are several context-dependent characteristics that are important to further interpret any particular interaction. According to Jordano [82, 83], any interaction is composed of 3 basic components: the co-occurrence, the encounter, and the outcome. To allow more efficient data aggregation and analysis, these 3 components should be properly documented. With that in mind, we propose a vocabulary that includes terms to capture specific details about these components, documenting the different contexts in which the interactions take place.

Although the interactions are being documented at the individual level, it does not require that the interacting organisms must be taxonomically identified at the species rank level. Following the DWC standard, organisms can be identified at any taxonomic rank (usually the lowest level taxonomic rank that can be determined). It is important since some studies do not identify the plant or pollinators at the species taxonomic rank. Some studies on pollination have identified organisms as functional groups, and despite the DWC standard not providing a specific term for documenting functional groups, it can be achieved by using the *remarks* terms in DWC (e.g., dwc:taxonRamarks, dwc:occurrenceRemarks) and providing the lowest taxonomic rank in the dwc:scientificName.

### The vocabulary

The plant–pollinator interactions vocabulary comprises a set of documents, including both machine- and human-readable forms. The vocabulary is composed of a list of terms, their definitions, usage comments, and examples, as well as a set of descriptive documents explaining how to use the vocabulary [58].

The terms in the vocabulary are divided into 6 categories: Animal, Plant, Flower, Interaction, Reproductive Success, and Nectar Dynamics. The categories are not formally defined as classes, and they are not part of the vocabulary; they are used only to organize the terms and to facilitate understanding by humans. The vocabulary includes a Term List (aka tdwgutility:TermList) with definitions of 48 terms represented as rdf:Property.

Each term in the vocabulary contains the following normative elements as defined in the TDWG SDS:

- **Term name:** a controlled value that represents the property or concept described by the term definition
- **Term label:** a word or short phrase that serves as a human-readable name for the term
- **Term IRI:** the HTTP Internationalized Resource Identifier (IRI) that uniquely identifies the current term
- **Definition:** the normative definition of the term, written in English
- **Modified:** the date in ISO 8601 date format on which the most recent version of the term was issued
- **Type:** the type of term (can be “Class,” “Property,” or “Concept”); in our case, all take the value “Property”

Additionally, for each term, we provide usage comments and examples as nonnormative content.

## Using Darwin Core to document biological interactions

The biodiversity community has found many different approaches to partially overcome the limitations of Darwin Core to share interactions data. Here we discuss the most common approaches, presenting their advantages and disadvantages.

### The “Association terms”

The DWC standard defines terms that can be used to document generic associations between instances of the *dwc:Occurrence* class and other resources (images, references, taxa, occurrences), which indicate that a resource was linked to a related resource of some type. Those terms are known as “Association terms” and include terms to document associations with taxa (dwc:associatedTaxa) and occurrences (dwc:associatedOccurrences). While the dwc:associatedTaxa term is meant to express an association of any kind (not only biological interactions) between an dwc:Occurrence and names of taxa, the term dwc:associatedOccurrences is meant to express an association between an dwc:Occurrence and 1 or more other dwc:Occurrence’s. The term wc:associatedTaxa is defined as “a list (concatenated and separated) of identifiers or names of taxa and the associations of this occurrence to each of them” (e.g., “pollinator of:” “*Fuchsia magellanica*”). However, the usage of this term is not well established, and we can find values with many different patterns in the available data (see Supplementary Material for a list of associations extracted from data sets in the GBIF registry). This approach is limited to documenting the taxonomic component of an interaction and does not allow capturing other interactions-related data or organism traits. Additionally, the nature of the association is often unspecified, or, when documented, it does not adopt any controlled vocabulary, which makes data less reusable and difficult to aggregate. In turn, using the term dwc:associatedOccurrences conveys similar problems, and therefore none of them are adequate to document biological interactions, since both lack many important data elements to correctly contextualize biological interactions (e.g., spatial and temporal information, interaction outcomes).

Using dwc:associatedTaxa is useful, for example, when one wants to document plant occurrences and the names of taxa visiting those particular plants, without documenting the individual visitors. However, due to the definition of the term dwc:associatedTaxa, we cannot assume that the association reported was observed as part of the same interaction event (e.g., the term may include 1 or more associations recorded at a previous time). A similar problem arises when using the dwc:associatedOccurrence term: we cannot assume that the location and the time documented for both associated occurrences are the same as those of the interaction, no matter how obvious it might be, since the definitions of the terms are too generic to support that assertion.

#### The dwc:ResourceRelationship class

The DWC standard also provides a solution to document any kind of relationship between records (e.g., occurrences, taxa, locations, identifications) in a more detailed way compared to the “Association terms.” The current version of DWC includes a comment in the description of the terms dwc:associatedTaxa and dwc:associatedOccurrences, which recommends the usage to dwc:ResourceRelationship class as an alternative to representing associations in more detail. Because of that, the dwc:ResourceRelationship class has been adopted by some initiatives and previous studies to document biological interactions [84, 85]. Although it allows documentation of biological interactions in more detail, there are some limitations to document characteristics of the relationships themselves (e.g., interaction outcomes).

Currently, DWC does not provide a solution for dwc:ResourceRelationship class using RDF, due to the way in which DWC “ID” terms are defined. Darwin Core contains a number of “ID” terms intended to designate identifiers (e.g., dwc:occurrenceID, dwc:identificationID, dwc:locationID). The “ID” terms observe 2 functions, specifying the class of the resource and indicating that the value of the term is an identifier. However, in RDF, these 2 functions are handled separately using rdf:type declarations and URIs for expressing the identifier of the subject resource. For most DWC “ID” terms, the Dublin Core dcterms:identifier can be used as a replacement to indicate the identifier of an RDF resource, but the same cannot be applied to dwc:ResourceRelationship. The dwc:ResourceRelationship class includes 2 “ID” terms: dwc:resourceID (“an identifier for the resource that is the subject of the relationship”) and dwc:relatedResourceID (“an identifier for a related resource; the object, rather than the subject of the relationship”). Because the definition of dcterms:identifier makes a clear assumption of its usage (“an unambiguous reference to the resource within a given context”), it is not clear whether dwc:ResourceRelationship would make sense in the context of RDF, as a dcterms:identifier would make no distinction between each term it serves as a replacement for (i.e., dwc:resourceID or dwc:relatedResourceID). The latest version of DWC includes the new ID term (dwc:relationshipOfResourceID), which has reactivated the discussion about representing the dwc:ResourceRelationship in RDF, but it is still under debate and beyond the scope of this article (see Baskauf and Webb [86] and New [87] for a discussion on this topic).

The “RDF world” is in its early stages of adoption by the biodiversity community [86]. However, there are some examples of dwc:ResourceRelationship usage to document biological interactions, as in the Catalogue of the Rust Fungi of Belgium [88] and in Plinian Core [85]. As the use of RDF grows, the RDF representation of biological interactions can be revised to properly meet the requirements of the linked open data principles.

It is relevant to mention that the adoption of dwc:ResourceRelationship as a solution for documenting and sharing species interactions by previous initiatives does not include the development and use of a common vocabulary. Instead, the dwc:ResourceRelationship is used only to link occurrences, taxa, and specimens but not to provide any data concerning the interaction itself (e.g., spatial and temporal information) or additional information related to the interaction.

#### Nonconventional approaches

There are also other terms that have been used to document biological interactions, like dwc:occurrenceRemarks (e.g., Scheinberg [89]) and dwc:dynamicProperties (e.g., Cheadle Center for Biodiversity and Ecological Restoration [90]). The problem is that these generic terms expect either free text content (e.g., dwc:occurrenceRemarks) or content to be captured in some format that is cumbersome for both the data publishers and users (e.g., dwc:dynamicProperties recommends formatting data using key:value encoding schemas).

The DWC standard does not include atomized terms for capturing interactions-related data or organisms traits, but it includes generic terms for documenting additional measurements, facts, characteristics, or assertions about a record (e.g., dwc:measurementType and dwc:measurementValue, included in the dwc:MeasurementOrFact class). Although less common than other approaches, we find examples of adoption of the dwc:MeasurementOrFact class to document biological interactions (e.g., [91]). Since the DWC standard itself does not contain controlled vocabularies of values for these terms, different communities have instead come up with some discipline-specific vocabularies, which are often not equivalent and difficult to compare to each other.

## The plant–pollinator interaction data model

The main concern with the previous approaches is that the interactions are not the central piece of information being documented; instead, they are treated as a link between occurrences or names of taxa. Since we are attempting to document interaction records between groups of organisms, the most appropriate approach is to represent any interaction as an instance of dwc:Event class. This approach is very similar to the one already being used in the GBIF network to document “sampling-event data” [92]. The main difference here is that, instead of sampling occurrences, we are documenting interactions between organisms and their occurrences.

In DWC, the dwc:Event class is defined as “an action that occurs at some location during some time” [93], which is particularly generic and encompasses the definitions of “intra-action” and “coaction” suggested by Lidicker [45]. Thus, these definitions support the adoption of the dwc:Event class to represent a biological interaction, and in the plant–pollinator interaction data model, the dwc:Event class is used to represent temporal and spatial details about the interactions. Therefore, a dwc:Event is linked to instances of dwc:Occurrence class representing the occurrences of the interacting organisms. In order to express the type and direction of the interactions, different approaches are taken depending on the implemented application schemas (DwC-Archive, XML, or RDF). Additionally, the dwc:MeasurementOrFact class should be used to represent any other characteristics of the interactions (e.g., outcomes) or occurrences (e.g., organism traits). The terms in the plant–pollinator interactions vocabulary should be used as values for dwc:measurementType. For those terms in the vocabulary that recommend the use of a controlled vocabulary, the dwc:measurementValue can be used referencing the appropriate controlled values.

Like in DWC, none of the terms are mandatory. Ideally, the more information provided, the better. However, depending on the amount of data collected or the specific objectives of the studies, only a portion of the terms can be filled. In order to make interaction data reusable in different contexts, a minimal set of terms must be used. Thus, every interaction must provide, at least, the following details:

- dwc:Occurrence: at least the taxonomic information must be present (dwc:scientificName)
- dwc:Event: at least the identifier of the event (dwc:eventID)
- dwc:ResourceRelationship: providing values for dwc:resourceID (the subject of the interaction), dwc:relatedResourceID (the object of the interaction), dwc:relantionshipOfResource or dwc:relantionshipOfResourceID (the interaction type; it can be very broadly defined such as *ecologically related to* or *participates in a biotic–biotic interaction with*), and for dwc:relationshipAccordingTo (the source of the interaction: person, organization, publication, or reference)

### Plant–pollinator interactions data as Darwin Core*–*Archive

Although documenting species interactions in text files and XML formats is less restrictive, the DwC-Archive [94] can be used to represent relations between resources (e.g., occurrence, taxon). While XML and RDF serialization formats can naturally handle one-to-many relations, the dwc:ResourceRelationship class and also the dwc:MeasurementOrFact class are of very limited use within Simple Darwin Core, as one-to-many relationships cannot be represented in “flat” files. The solution adopted by the DwC-Archive is to use a star schema, where a “core table” is linked to many “extension tables” by means of a unique identifier assigned to each record in the core table (i.e., the core id). Thus, it is possible to have multiple records in the extension tables referencing one record in the core table (i.e., implementing a one-to-many relationship).

For the scope of plant–pollinator interaction data, we have extended the well-known “sampling event data model.” In the extended model, similar to the original model, the “core table” in the DwC-Archive represents events (instances of dwc:Event class), and an “extension table” is used to record the occurrences (instances of dwc:Occurrence class) related to each event in the core table. The difference is that, in the extended model, multiple characteristics of each event should be documented using either the dwc:MeasurementOrFact class or obis:ExtendedMeasurementOrFact (eMoF) extension [95].

The MoF class can be used to express one-to-many relationships of features associated with the target events (interactions) represented with the dwc:Event class (i.e., depicting the characteristics of the interactions). However, it cannot be used directly to document a characteristic of a plant or an animal participating in a particular interaction when DwC-Archives are used to standardize data due to limitations of the star schema. Although some workarounds have been attempted to overcome this, they are cumbersome for both data providers and users.

Otherwise, the eMoF extension was specially designed to handle environmental data in conjunction with species occurrence data. The eMoF extension is built on the existing dwc:MeasurementOrFact, using dwc:occurrenceID and adding 3 new terms: obis:measurementTypeID, obis:measurementValueID, and obis:measurementUnitID. The dwc:occurrenceID term is used to circumvent the limitations of the star schema and link measurement records in the obis:ExtendedMeasurementOrFact extension to occurrence records in the dwc:Occurrence extension (Fig. 2). The other 3 terms are used to constrain and standardize the measurement types, values, and units since the dwc:MeasurementOrFact terms are completely unconstrained and can be populated with free text content. As stated by the authors, “The three new terms should be populated using controlled vocabularies referenced using URIs)” [95].

**Figure 1:**
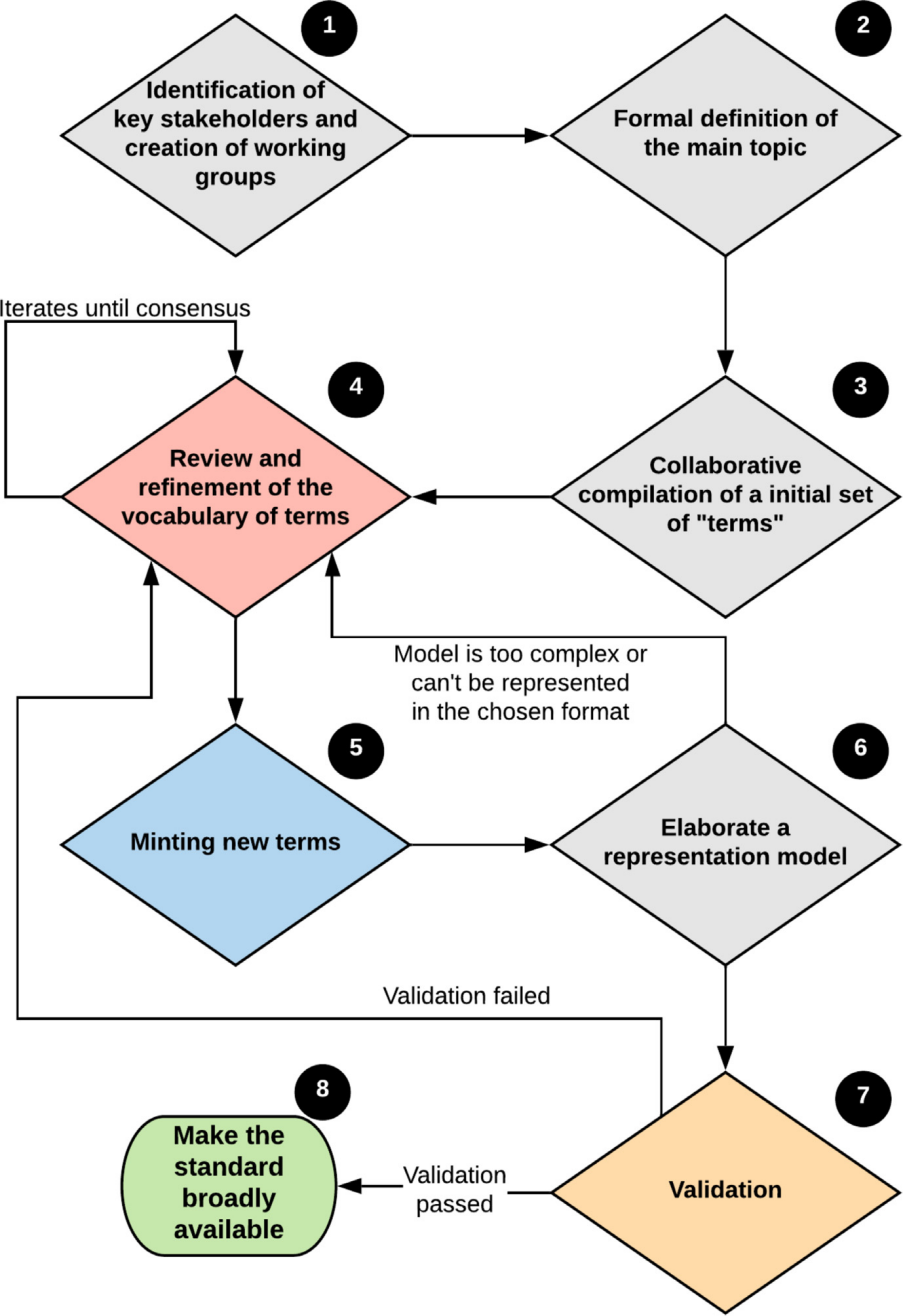
Suggestion of a community workflow to create a new vocabulary of terms. Numbers in the black circles indicate the steps described above in the text.

**Figure 2:**
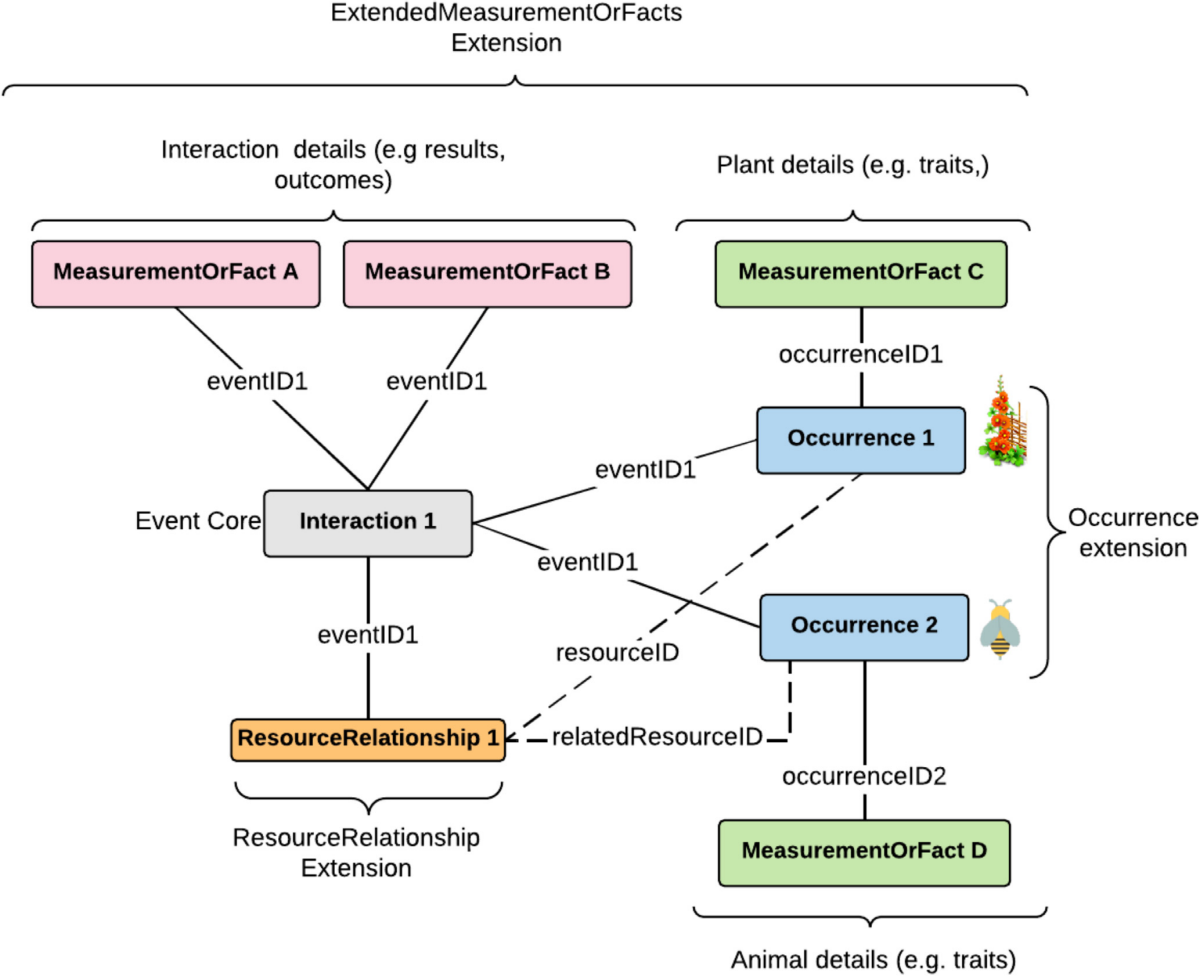
Overview of the data schema to represent plant–pollinator interactions. The plant and animal occurrences (blue boxes) are linked to the interaction (dwc:Event, gray box) using the dwc:eventID (full lines). Measurements related to the interactions (MeasurementOrFact A and MeasurementOrFact B, pink box, e.g., ppi:resourceCollected, ppi:nectarCollectingBodyPart, ppi:numberOfRemovedPollenGrains) are linked directly to the interaction (gray box) using the dwc:eventID. Measurements related to the occurrences (MeasurementOrFact C and MeasurementOrFact D, green boxes, e.g., ppi:flowerColor, ppi:floralAttractants, ppi:caste) are linked to the interactions using the dwc:eventID and the dwc:occurrenceID fields of the obis:ExtendedMeasurementOrFact extension (dashed lines). The direction and the type of the interaction are given by the dwc:ResourceRelationship class (orange box), linked directly to the interaction using dwc:eventID (full lines) and indirectly to the occurrences using dwc:resourceID and dwc:relatedResourceID terms (dashed lines).

Thus, when using eMoF, the terms in the plant–pollinator interactions vocabulary should be used as values for obis:measurementTypeID and the obis:measurementValueID can be used referencing terms in controlled vocabularies providing the appropriate URI. The classic dwc:measurementType and dwc:measurementValue should be used to capture human-readable representations of the values used in the corresponding ID fields.

Additionally, in the extended model, the dwc:ResourceRelationship class is used to document the relationships between the occurrences. The dwc:ResourceRelationship class allows documentation of the type of the interaction (dwc:relationshipOfResource and dwc:relationshipOfResourceID) and the direction of the interaction, since the dwc:ResourceRelationship has terms for the subject (dwc:resourceID) and object (dwc:relatedResourceID) of the relationship (dwc:resourceRelationshipID). For the type of the interaction, it is recommended to use values from the Relations Ontology [72]. The Plant–Pollinator Interactions vocabulary includes a guide explaining how to document plant–pollinator interactions using DwC-Archive schema [96].

### Plant–pollinator interactions data as XML

The implementation using XML is very similar to the implementation using DwC-Archive. The main difference is that in XML, we do not have the limitations of the star schema, and one-to-many relationships can be handled naturally. When using XML, we do not need to use obis:ExtendedMeasurementOrFact to document additional characteristics of the dwc:Occurrences. Instead, the dwc:MeasurementOrFact class should be used providing the appropriate dwc:measurementID in the dwc:Occurrence. Because the DWC XML schema does not define any constraint on the duplication of ID terms inside an XML element representing a DWC class [97], we can document multiple instances of the dwc:MeasurementOrFact class and then refer to them in dwc:Occurrence or dwc:Event instances using the dwc:measurementID term as a link between the records. A guide explaining how to document plant–pollinator interactions using XML is provided alongside the vocabulary [98].

### Plant–pollinator interactions data as RDF

Representing interactions, or any relations, in DWC using RDF is limited due to the reasons presented before. However, we can still document interactions in RDF according to the data model proposed, if an additional RDF vocabulary is provided. The Darwin-SW (dsw; Baskauf and Webb [86]) is an RDF vocabulary designed to complement the Darwin Core Standard and, when used in conjunction with Darwin Core IRI terms (dwciri), allows one to document biological interactions using RDF.

**Figure 3:**
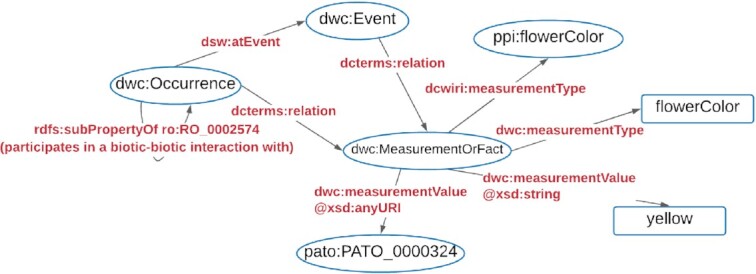
Simplified diagram of a graph structure that can be used to represent biological interactions in conjunction with plant–pollinator interactions vocabulary using RDF. Note that the diagram shows links between instances of classes, but for simplicity, only the class URIs of those instances are indicated in the ovals. dsw:atEvent is an abbreviation for http://purl.org/dsw/atEvent; ppi:flowerColor is an abbreviation for http://rs.rebipp.org.br/ppi/flowerColor; dcterms:relation is an abbreviation for http://purl.org/dc/terms/relation; dwc: and dwciri: are abbreviations for http://rs.tdwg.org/dwc/terms/ and http://rs.tdwg.org/dwc/iri/, respectively.

In RDF, the interactions are still represented using the dwc:Event class, but the link between instances of the dwc:Occurrence class and the dwc:Event class are made using the dsw:atEvent term as predicate in the RDF triplet, having an instance of dwc:Occurrence as the subject and an instance of dwc:Event as the object (Fig. 3). The type and direction of an interaction are given naturally by the RDF triplet composed by 2 instances of the dwc:Occurrence class as subject and object, as well as a term from the Relations Ontology as predicate (Fig. 3).

Additionally, the dwc:MeasurementOrFact class should be used to document any other characteristics of the interactions or the occurrences using terms from the plant–pollinator vocabulary to specify the properties dwciri:measurementType (for nonliteral objects) and dwc:measurementType (for literal objects). For terms in the plant–pollinator vocabulary that recommend the adoption of a controlled vocabulary, the term dwc:measurementValue can be used with literal objects providing xsd:string as the rdf:datatype attribute of the dwc:measurementValue. Alternatively, a more appropriate solution is to use a nonliteral object (URI of the term in the controlled vocabulary) with xsd:anyURI as the rdf:datatype attribute. Instances of dwc:Event and dwc:Occurrence classes are then linked to the instances of dwc:MeasurementOrFact using the term dcterms:relation from Dublin Core (purl.org/dc/terms/), following the Darwin Core RDF Guide [99]. The Plant–Pollinator Interactions vocabulary includes a guide explaining how to document plant–pollinator interactions using RDF [100].

Although we recommend the adoption of Relations Ontology terms as predicates for documenting interactions, terms from other vocabularies and ontologies can be used as well. Similarly, terms from other vocabularies can be used to document any additional measurements or facts about the interactions and occurrences.

## Documenting plant–pollinator interaction networks

Plant–pollinator interactions are widely studied using complex interaction networks [101, 102]. Interaction networks are usually documented as adjacency (binary or not) matrices, where nodes represent the species and the edges represent the interactions between nodes. Although, prior to the construction of such networks, the interactions are sampling in field or inferred from pollen grains obtained from the animal’s body, most of the plant–pollinator interaction data found in the literature are summarized as interaction networks.

In this particular case, the plant–pollinator schema and vocabulary presented here can also be used to document the interactions at the record level. The solution is to document each edge (i.e., interaction) in the network as a dwc:Event in the proposed schema. The dwc:Occurrence does not require specifying the number of individuals participating in the interaction, and each node in the network can be documented as a dwc:Occurrence of a particular taxon (since the interaction networks have defined spatial and temporal scopes).

Since the interaction networks have the minimal information needed to represent an interaction in the proposed model, there is no impediment to document them in same way as other use cases.

## Conclusions

By presenting a vocabulary of terms to assist digitization, sharing, aggregation, and use of plant–pollinator interactions data, this work provides the means to represent such data in a more complete, accurate, and standardized way. This vocabulary can contribute to overcoming some of the technical limitations that have hindered the analyses of large quantities of available data, thus helping to fill an important, long-standing knowledge gap. The process required the involvement and effort of a diverse group of specialists during a period of 5 years and has provided a great opportunity to gain experience around community-driven vocabulary building, which we share for future initiatives.

Although this model was conceived for plant–pollinator interactions, we expect that it can be adopted as a general framework for different types of ecological interactions along the antagonism–mutualism continuum, by providing appropriate controlled vocabularies for dwc:measurementType and obis:measurementTypeID.

The plant–pollinator interactions vocabulary was designed to be fully compatible with the Darwin Core Standard, so that data that use it can be easily aggregated by current practices of biodiversity data portals. Despite data portals like GBIF not indexing all terms contained in data sets (e.g., measurement or facts), and therefore direct searches for some terms are not always possible, the data are still broadly and openly available in a standardized form and can be retrieved from those same portals. To explore and to take full advantage of plant–pollinator standardized data, we are developing an information system and a database of plant–pollinator interactions, as part of the Safeguarding Pollinators and Pollination Services (SURPASS) project [103]. The system will be publicly available on the REBIPP website and function as a data portal of plant–pollinator interactions integrating the biodiversity network for sharing data among member nodes. The system will allow searching for specific interactions records using terms in the plant–pollinator vocabulary for filtering data. The open data policy will guarantee access to plant–pollinator standardized data to many communities around the globe.

This work is also aligned with and part of the Biological Interactions Data Interest Group of the Biodiversity Information Standards [104] organization, which aims to standardize biological interactions data, and we expect the plant–pollinator interactions vocabulary to help achieve this goal. The adoption of a standard depends on how well it can accommodate a wide gamut of use cases without overcompromising its simplicity. Thus, the participation of multiple international actors from the pollination biology and biodiversity informatics communities, gathered around TDWG, was essential for a solution that satisfies, as much as possible, the needs and expectations of most potential stakeholders and helps to advance science in this field. We expect that the plant–pollinator interaction standard and the information system will enable data aggregation from a variety of sources worldwide at higher levels than we have experienced so far, significantly amplifying the plant–pollinator data availability for global synthesis and contributing to the knowledge base that allows developing responsible ecosystem restoration, biodiversity conservation, and sustainable agriculture.

## Data Availability

The plant–pollinator interactions vocabulary presented in this article is available in the GitHub repository, https://github.com/rebipp/ppi.

## Additional files

**associatedTaxa_supplementary_material.txt**

**ppi_terms_versions_supplementary_material.xlsx**

giac043_GIGA-D-22-00029_Original_Submission

giac043_GIGA-D-22-00029_Revision_1

giac043_Response_to_Reviewer_Comments_Original_Submission

giac043_Reviewer_1_Report_Original_Submission Pedro Jordano -- 3/15/2022 Reviewed

giac043_Reviewer_2_Report_Original_Submission Daichi Funamoto -- 3/24/2022 Reviewed

giac043_Supplemental_Files

## Abbreviations

ABCD: Access to Biological Collection Data; DWC: Findable, Accessible, Interoperable, and Reusable data; DwC-Archive: Darwin Core Archive; EML: Ecological Metadata Language; eMoF: extended measurement or fact; EOL: Encyclopedia of Life; FAIR: Findable, Accessible, Interoperable, and Reusable; FAO: Food and Agriculture Organization of the United Nations; GBIF: Global Biodiversity Information Facility; GEF: Global Environment Facility; GloBI: Global Biotic Interactions; GoMexSI: Gulf of Mexico Species Interaction; HTTP: Hypertext Transfer Protocol; IABIN-PTN: InterAmerican Biodiversity Information Network–Pollinators Thematic Network; IPBES: Intergovernmental Science–Policy Platform on Biodiversity and Ecosystem Services; IRI: Internationalized Resource Identifier; ISO: International Organization for Standardization; IWDB: Interaction Web DataBase; MoF: measurement or fact; RDF: Resource Description Framework; REBIPP: Brazilian Network of Plant–Pollinator Interactions; SDS: TDWG Standards Documentation Standard; SURPASS: Safeguarding Pollinators and Pollination Services; TDWG: Biodiversity Information Standards; UNEP: United Nations Environment Programme; URI: Uniform Resource Identifier; USGS: United States Geological Survey; XML: eXtensible Markup Language.

## Competing interests

The author(s) declare that they have no competing interests.

## Funding

This work was funded by the “São Paulo Research Foundation” (FAPESP) within the project “Safeguarding Pollination Services in a Changing World: Theory into Practice” (SURPASS2). FAPESP:2018/14994-1. PI: Antonio Mauro Saraiva. The funder had no role in conducting the research and/or during the preparation of the article.

## Authors' contributions

JAS, PFZ, AMS, KA, EAC, AKV, TCG, and JSS conceptualized the study. AMS, KA, and PFZ provided supervision. AMS and KA provided project administration and acquired funding. JAS, PFZ, AMS, DPD, FMS, PJB provided formal analysis. JAS, AMS, PFZ, DPD, FMS, KA, and AKV provided methodology. JAS, AMS, PFZ, DPD, FMS, KA, MW, IGV, LF, MMM, and ARR wrote the original draft of the manuscript. All authors provided investigation and review and edited the final manuscript.

## References

[1] Potts SG, Imperatriz-Fonseca V, Ngo HT, et al. Safeguarding pollinators and their values to human well-being. Nature. 2016;540(7632):220–9. 27894123 10.1038/nature20588

[2] Ollerton J, Winfree R, Tarrant S. How many flowering plants are pollinated by animals?. Oikos. 2011;120(3):321–6.

[3] Klein AM, Vaissière BE, Cane JH, et al. Importance of pollinators in changing landscapes for world crops. Proc R Soc B Biol Sci. 2007;274(1608):303–13. 10.1098/rspb.2006.3721 PMC1702377 17164193

[4] Tylianakis JM, Didham RK, Bascompte J, et al. Global change and species interactions in terrestrial ecosystems. Ecol Lett. 2008;11(12):1351–63. 19062363 10.1111/j.1461-0248.2008.01250.x

[5] Rodger JG, Bennett JM, Razanajatovo M, et al. Widespread vulnerability of flowering plant seed production to pollinator declines. Sci Adv. 2021;7(42):eabd3524. 34644118 10.1126/sciadv.abd3524 PMC8514087

[6] CDB . Report of the Sixth Meeting of the Conference of the Parties to the Convention on Biological Diversity (UNEP/CBD/COP/20/Part 2) Agricultural Biological Diversity Decision VI/5. The Hague; 2002.CONVENTION ON BIOLOGICAL DIVERSITY

[7] Schmeller DS, Bridgewater P. The Intergovernmental Platform on Biodiversity and Ecosystem Services (IPBES): progress and next steps. Biodiversity Conservation. 2016;25(5):801–5. 10.1007/s10531-022-02500-y PMC9643941 36405572

[8] IPBES , SG Pottsz, VL Imperatriz-Fonseca, HT Ngo 552 The assessment report of the Intergovernmental Science-Policy Platform on Biodiversity and Ecosystem Services on pollinators, pollination and food production. Secretariat of the Intergovernmental Science-Policy Platform on Biodiversity and Ecosystem Services; 2016. Bonn, Germany. 10.5281/zenodo.3402857.

[9] Dias B, Raw A, Fonseca V. International Pollinators Initiative: The São Paulo Declaration on Pollinators. Report on th Recommendations of the Workshop on the Conservation and Sustainable Use of Pollinators in Agriculture with Emphasis on Bees. 66 Brazilian Ministry of the Environment (MMA); Brasília, Brazil; 1999.

[10] Food and Agriculture Organization of the United Nations . FAO’s global action on pollination services for sustainable agriculture. https://www.fao.org/pollination/en/.

[11] U.S. Geological Survey . Pollinator conservation. https://www.usgs.gov/centers/eesc/science/pollinator-conservation.

[12] European Commission . EU pollinators. https://ec.europa.eu/environment/nature/conservation/species/pollinators/index_en.htm.

[13] Wolowski M, Ashman TL, Freitas L. Meta-analysis of pollen limitation reveals the relevance of pollination generalization in the Atlantic forest of Brazil. PLoS ONE. 2014;9(2):e89498. 24586827 10.1371/journal.pone.0089498 PMC3931788

[14] Menz MHM, Phillips RD, Winfree R, et al. Reconnecting plants and pollinators: challenges in the restoration of pollination mutualisms. Trends Plant Sci. 2011;16(1):4–12. 20980193 10.1016/j.tplants.2010.09.006

[15] Saraiva AM, Imperatriz-Fonseca VL, Cunha RS, et al., 1:WebBee–a web-based information network on bees. Revista de Engenharia de Computação e Sistemas Digitais. 2003;(1):77–86.

[16] Redhead JW, Coombes CF, Dean HJ, et al. Plant-Pollinator Interactions Database for Construction of Potential Networks. NERC Environmental Information Data Centre; 2018. 10.5285/6d8d5cb5-bd54-4da7-903a-15bd4bbd531b.

[17] Conservation Center for Plant Plant-Pollinator Interaction Explorer. 2020. https://plant-pollinator.shinyapps.io/shinyapp/. Accessed: 12 May, 2022.

[18] Poelen JH, Simons JD, Mungall CJ. Global biotic interactions: an open infrastructure to share and analyze species-interaction datasets. Ecol Inform. 2014;24:148–59.

[19] Simons JD, Yuan M, Carollo C, et al. Building a fisheries trophic interaction database for management and modeling research in the Gulf of Mexico large marine ecosystem. Bull Marine Sci. 2013;89(1):135–60.

[20] Poisot T, Baiser B, Dunne JA, et al. mangal - making ecological network analysis simple. Ecography. 2015: 39:384–90. 10.1111/ecog.00976.

[21] Interaction Web DataBase . http://www.ecologia.ib.usp.br/iwdb/.

[22] Beas-Luna R, Novak M, Carr MH, et al. An online database for informing ecological network models. PLoS ONE. 2014;9(10):e109356. http://kelpforest.ucsc.edu. 25343723 10.1371/journal.pone.0109356 PMC4208745

[23] LIFEWEBS PROJECT . http://www.lifewebs.net/.

[24] Thompson RM, Brose U, Dunne JA, et al. Food webs: reconciling the structure and function of biodiversity. Trends Ecol Evol. 2012;27(12):689–97. 22959162 10.1016/j.tree.2012.08.005

[25] Fortuna MA, Ortega R, Bascompte J. The Web of Life. 2014; arXiv. 10.48550/arxiv.1403.2575.

[26] Hortal J, de Bello F, Diniz-Filho JAF, et al. Seven shortfalls that beset large-scale knowledge of biodiversity. Annu Rev Ecol Evol Systematics. 2015;46(1):523–49.

[27] Vizentin-Bugoni J, Maruyama PK, de Souza CS, et al. Plant-pollinator networks in the tropics: a review. In: Dáttilo W, Rico-Gray V, eds. Ecological Networks in the Tropics: An Integrative Overview of Species Interactions from Some of the Most Species-Rich Habitats on Earth. Cham, Switzerland: Springer; 2018:73–91.

[28] Schemske DW, Mittelbach GG, Cornell HV, et al. Is there a latitudinal gradient in the importance of biotic interactions?. Annu Rev Ecol Evol Systematics. 2009;40(1):245–69.

[29] Arzabe AA, Aguirre LF, Baldelomar MP, et al. Assessing the geographic dichotomy hypothesis with cacti in South America. Plant Biol. 2018;20(2):399–402. 29156089 10.1111/plb.12669

[30] Tilman D. Functional diversity. In: Levin SA, ed. Encyclopedia of Biodiversity. New York: Elsevier; 2001:109–20.

[31] Emer C, Galetti M, Pizo MA, et al. Defaunation precipitates the extinction of evolutionarily distinct interactions in the Anthropocene. Sci Adv. 2019;5(6):eaav6699. 31223648 10.1126/sciadv.aav6699 PMC6584213

[32] Mouillot D, Bellwood DR, Baraloto C, et al. Rare species support vulnerable functions in high-diversity ecosystems. PLoS Biol. 2013;11(5):e1001569. 23723735 10.1371/journal.pbio.1001569 PMC3665844

[33] Allen-Perkins A, Magrach A, Dainese M, et al. CropPol: A Dynamic, Open and Global Database on Crop Pollination. Ecology. 2022;103(3):e3614. 10.1002/ecy.3614. 34921678

[34] Pimm SL, Lawton JH, Cohen JE. Food web patterns and their consequences. Nature. 1991;350(6320):669–74.

[35] Wilkinson MD, Dumontier M, Aalbersberg IJ, et al. The FAIR guiding principles for scientific data management and stewardship. Sci Data. 2016;3(1):160018. 26978244 10.1038/sdata.2016.18 PMC4792175

[36] Wieczorek J, Bloom D, Guralnick R, et al. Darwin Core: an evolving community-developed biodiversity data standard. PLoS ONE. 2012;7(1):e29715. 22238640 10.1371/journal.pone.0029715 PMC3253084

[37] GBIF: The Global Biodiversity Information Facility . What is GBIF? 2022. https://www.gbif.org/what-is-gbif.

[38] Access to Biological Collection Data Task Group . Access to Biological Collection Data (ABCD), Version 2.06. Biodiversity Information Standards (TDWG) 2007.; http://www.tdwg.org/standards/115

[39] GBIF/TDWG Multimedia Resources Task Group . Audubon Core Multimedia Resources Metadata Schema: Biodiversity Information Standards (TDWG). 2013. http://www.tdwg.org/standards/638.

[40] Jones M, O’Brien M, Mecum B, et al. Ecological Metadata Language version 2.2.0. 2019. https://eml.ecoinformatics.org.

[41] Droege G, Barker K, Seberg O, et al. The Global Genome Biodiversity Network (GGBN) data standard specification. Database. 2016;2016:baw125. 27694206 10.1093/database/baw125 PMC5045859

[42] Endresen DTF, Knüpffer H. The Darwin Core Extension for genebanks opens up new opportunities for sharing Genebank datasets. Biodiversity Informatics. 2012;8. 10.17161/bi.v8i1.4095.

[43] Brenskelle L, Wieczorek J, Davis E, et al. A community-developed extension to Darwin Core for reporting the chronometric age of specimens. bioRxiv 2021. 10.1101/2021.11.24.469822. PMC9477364 36107867

[44] GBIF Registered Extensions. https://tools.gbif.org/dwca-validator/extensions.do.

[45] Lidicker WZ. A clarification of interactions in ecological systems. BioScience. 1979;29(8):475–7.

[46] Global Biotic Interactions. Models in fashion. https://www.globalbioticinteractions.org/2018/08/16/models-in-fashion/#darwin-core-archive--encyclopedia-of-life-flavor.

[47] Ruggiero M, Saraiva AM. A pollinators thematic network for the Americas. In: The Proceedings of TDWG. TDWG Bratislava, Slovakia: 2007.

[48] TDWG Wiki Archive . https://github.com/tdwg/wiki-archive/blob/d77f897a52d96f1bd974d5c438790017b8419fac/twiki/data/DarwinCore/InteractionExtension.txt.

[49] Saraiva AM, Cartolano Júnior EA, De Giovanni R, et al. Exchanging specimen interaction data using Darwin Core. In: The Proceedings of TDWG. Biodiversity Information Standards (TDWG) 68–69. Montpellier, France: 2009.

[50] Cartolano Júnior EA. Proposta de um sistema de informação orientado a serviços sobre a biodiversidade de abelhas. São Paulo, Brazil: São Paulo, Brazil. Dissertação (Mestrado em Sistemas Digitais) - Escola Politécnica Universidade de São Paulo; 2009. 10.11606/D.3.2009.tde-23092009-151526

[51] Saraiva AM, Gemmill-Herren B, Ruggiero M. A common schema for managing plant-pollinator interaction data. Report on Progress for GEF/UNEP/FAO Project: “Conservations and Management of Pollinators for Sustainable Agriculture, through an Ecosystem Approach.” 2010: United Nations Environment Programme (UNEP) 33–33.

[52] Carvalheiro LG, Saraiva AM, Giannini TC. Establishing knowledge management systems for ecological interactions: The Case of Crop Pollinators. In: Gemmil-Herren Barbara Pollination Services to Agriculture. New YorkRoutledge 2016:92–112.

[53] Tremblay MS, Aubert S, Barnes JD, et al. Sedentary Behavior Research Network (SBRN): terminology consensus project process and Outcome. Int J Behav Nutr Phys Act. 2017;14(1):75. 28599680 10.1186/s12966-017-0525-8 PMC5466781

[54] Zeng ML, Chan LM. Metadata interoperability and standardization: a study of methodology, part II: achieving interoperability at the record and repository levels. D Lib Mag. 2006;12. (6):1082–9873.

[55] Duval E, Hodgins W, Sutton SA, et al. Metadata principles and practicalities. D Lib Mag. 2002;8:1–10.

[56] Pomerantz J. Metadata. Cambridge, MA: MIT Press; 2015.

[57] Issues: rebipp/ppi. https://github.com/rebipp/ppi.

[58] REBIPP . Plant-pollinator interactions vocabulary quick reference guide. https://ppi.rebipp.org.br/terms/.

[59] REBIPP . Plant-pollinator interactions controlled vocabulary list of terms. https://ppi.rebipp.org.br/cv/.

[60] Vocabulary Maintenance Specification Task Group . Vocabulary Maintenance Standard. Biodiversity Information Standards (TDWG). http://www.tdwg.org/standards/642 2017.

[61] Vocabulary Maintenance Specification Task Group . Standards Documentation Standard. Biodiversity Information Standards (TDWG). http://www.tdwg.org/standards/147 2017.

[62] GitHub. https://github.com.

[63] Darwin Core quick reference guide. https://dwc.tdwg.org/terms/.

[64] Chapman A, Belbin L, Zermoglio P, et al. Developing standards for improved data quality and for selecting fit for use biodiversity data. Biodiversity Inform Sci Standards. 2020;4:e50889.

[65] Cooper L, Meier A, Laporte MA, et al. The Planteome Database: an integrated resource for reference ontologies, plant genomics and phenomics. Nucleic Acids Res. 2018;46(D1):D1168–80. 29186578 10.1093/nar/gkx1152 PMC5753347

[66] PATO: The Phenotype And Trait Ontology. https://github.com/pato-ontology/pato.

[67] Hoehndorf R, Alshahrani M, Gkoutos GV, et al. The Flora Phenotype Ontology (FLOPO): tool for integrating morphological traits and phenotypes of vascular plants. J Biomed Semantics. 2016;7(1):65. 27842607 10.1186/s13326-016-0107-8 PMC5109718

[68] Yoder MJ, Mikó I, Seltmann KC, et al. A gross anatomy ontology for hymenoptera. PLoS ONE. 2010;5(12):e15991. 21209921 10.1371/journal.pone.0015991 PMC3012123

[69] Park CA, Bello SM, Smith CL, et al. The vertebrate trait ontology: a controlled vocabulary for the annotation of trait data across species. J Biomed Semantics. 2013;4(1):13. 23937709 10.1186/2041-1480-4-13 PMC3851175

[70] Mungall CJ, Torniai C, Gkoutos GV, et al. Uberon, an integrative multi-species anatomy ontology. Genome Biol. 2012;13(1):R5. 22293552 10.1186/gb-2012-13-1-r5 PMC3334586

[71] Buttigieg PL, Morrison N, Smith B, et al. The environment ontology: contextualising biological and biomedical entities. J Biomed Semantics. 2013;4(1):43. 24330602 10.1186/2041-1480-4-43 PMC3904460

[72] Smith B, Ceusters W, Klagges B, et al. Relations in biomedical ontologies. Genome Biol. 2005;6(5):R46. 15892874 10.1186/gb-2005-6-5-r46 PMC1175958

[73] Bronstein JL. Mutualism. Oxford, UK: Oxford University Press; 2015.

[74] Pedruski MT, Fussmann GF, Gonzalez A. Predicting the outcome of competition when fitness inequality is variable. R Soc Open Sci. 2015;2(8):150274. 10.1098/rsos.150274 26361557 PMC4555862

[75] Mazancourt CD, Loreau M, Dieckmann U. Understanding mutualism when there is adaptation to the partner. J Ecol. 2005;93(2):305–14.

[76] Martin BD, Schwab E. Current usage of symbiosis and associated terminology. Int J Biol. 2012;5(1):32.

[77] Cassidy C, Grange LJ, Garcia C, et al. Species interactions and environmental context affect intraspecific behavioural trait variation and ecosystem function. Proc R Soc B Biol Sci. 2020;287(1919):20192143. 10.1098/rspb.2019.2143 PMC7015344 31992167

[78] Werner EE . Individual behavior and higher-order species interactions. Am Natural. 1992;140:S5–S32.

[79] Brosi BJ . Pollinator specialization: from the individual to the community. New Phytologist. 2016;210(4):1190–4. 27038018 10.1111/nph.13951

[80] Nakazawa T . Species interaction: revisiting its terminology and concept. Ecol Res. 2020;35(6):1106–13.

[81] Ontobee. OBI—class: organism. http://www.ontobee.org/ontology/OBI?iri=http://purl.obolibrary.org/obo/OBI_0100026.

[82] Jordano P . The biodiversity of ecological interactions: challenges for recording and documenting the web of life. Biodiversity Inform Sci Standards. 2021;5:e75564.

[83] Jordano P . Sampling networks of ecological interactions. Functional Ecol. 2016;30(12):1883–93.

[84] Cartolano EA Jr, Saraiva AM, Correa PLP, et al. Uma Proposta de Esquema de Dados de Relacionamento Entre Espécimes. In: XXXIII Conferencia Latinoamericana de Informática. Anais do XXXIII CLEI, 2007, 2007:1–8. San Jose: Costa Rica.

[85] Pando F . How species interactions are managed in Plinian Core: status and questions. Biodiversity Inform Sci Standards. 2017;1:e20556.

[86] Baskauf SJ, Webb CO. Darwin-SW: Darwin Core-based terms for expressing biodiversity data as RDF. Semantic Web. 2016;7(6):629–43.

[87] New Term—relationshipOfResourceID · Issue #283 · tdwg/dwc. https://github.com/tdwg/dwc/issues/283.

[88] Vanderweyen A, Fraiture A, Groom Q, et al. Catalogue of the Rust Fungi of Belgium 2019; Version 1.2. Meise Botanic Garden: Checklist dataset. 10.15468/2dboyn.

[89] Scheinberg L. CAS Herpetology (HERP) 2019; Version 33.33. California Academy of Sciences: Occurrence dataset. 10.15468/bvoyqy.

[90] Cheadle Center for Biodiversity and Ecological Restoration . University of California Santa Barbara Invertebrate Zoology Collection. 2021. 10.15468/w6hvhvcessed%via%GBIF.org.

[91] Faulwetter S, Markantonatou V, Pavloudi C, et al. Polytraits: a database on biological traits of marine polychaetes. Biodiversity Data J. 2014;2:e1024. 10.3897/BDJ.2.e1024 PMC4030217 24855436

[92] GBIF. Introduction to sampling-event data. https://www.gbif.org/sampling-event-data.

[93] Darwin Core Maintenance Group. List of Darwin Core terms. Biodiversity Information Standards (TDWG). 2021. http://rs.tdwg.org/dwc/doc/list/2021-07-15.

[94] GBIF. Darwin Core Archive Assistant v1.1. http://tools.gbif.org/dwca-assistant/.

[95] De Pooter D, Appeltans W, Bailly N, et al. Toward a new data standard for combined marine biological and environmental datasets: expanding OBIS beyond species occurrences. Biodiversity Data J. 2017;5:e10989. 10.3897/BDJ.5.e10989 PMC5345125 28325978

[96] REBIPP. Plant-pollinator interactions vocabulary text guide. https://ppi.rebipp.org.br/text/.

[97] Darwin Core XSD Schema. https://github.com/tdwg/dwc/blob/master/docs/xml/tdwg_dwcterms.xsd.

[98] REBIPP. Plant-pollinator interactions vocabulary XML guide. https://ppi.rebipp.org.br/xml/.

[99] Groups Darwin Core and RDF/OWL Task . Darwin Core RDF guide, Biodiversity Information Standards (TDWG);2015. http://rs.tdwg.org/dwc/terms/guides/rdf/2021-07-15.

[100] REBIPP. Plant-pollinator interactions vocabulary RDF guide. https://ppi.rebipp.org.br/rdf/.

[101] Bascompte J. Mutualistic networks. Frontiers in Ecology and the Environment. 2009; 7(8):429–36. 10.1890/080026.

[102] Memmott J. The structure of a plant-pollinator food web. Ecology Letters. 2(5):1999:276–80. 10.1046/j.1461-0248.1999.00087.x. 33810635

[103] SURPASS2. https://bee-surpass.org/.

[104] TDWG. Biological interactions data. https://www.tdwg.org/community/interaction/.

