# Supplementary material for: Data standardization of plant–pollinator interactions
Source: Gigascience. 2022 May 26;11:giac043. doi: 10.1093/gigascience/giac043 (PMC9154084; doi:10.1093/gigascience/giac043)
Supplement: giac043_GIGA-D-22-00029_Revision_1 [file giac043_giga-d-22-00029_revision_1.pdf]

GigaScience

Data standardization of plant-pollinator interactions

--Manuscript Draft--

**Manuscript Number:**	GIGA-D-22-00029R1
**Full Title:**	Data standardization of plant-pollinator interactions
**Article Type:**	Review
**Funding Information:**	Fundação de Amparo à Pesquisa do Estado de São Paulo (2018/14994-1) — Mr. Antonio M Saraiva
**Abstract:**	Background: animal pollination is an important ecosystem function and service, ensuring both the integrity of natural systems and human well-being. Although many knowledge shortfalls remain, some high-quality datasets on biological interactions are now available. The development and adoption of standards for biodiversity data and metadata has promoted great advances in biological data sharing and aggregation, supporting large scale studies and science-based public policies. However, these standards are currently not suitable to fully support interaction data sharing. Results: here we present a vocabulary of terms and a data model for sharing plant-pollinator interactions data based on the Darwin Core standard. The vocabulary introduces 48 new terms targeting several aspects of plant-pollinator interactions, and can be used to capture information from different approaches and scales. Additionally, we provide solutions for data serialization using RDF, XML and DwC-Archives and recommendations of existing controlled vocabularies for some of the terms. Our contribution supports open access to standardized data on plant-pollinator interactions. Conclusion: the adoption of the vocabulary would facilitate data sharing to support studies ranging from the spatial and temporal distribution of interactions, to the taxonomic, phenological, functional, and phylogenetic aspects of plant-pollinator interactions. We expect to fill data and knowledge gaps, thus further enabling scientific research on the ecology and evolution of plant-pollinator communities, biodiversity conservation, ecosystem services, and the development of public policies. The proposed data model is flexible and can be adapted for sharing other types of interactions data by developing discipline-specific vocabularies of terms.
**Corresponding Author:**	José Augusto Salim<br>Universidade Estadual de Campinas<br>Campinas, São Paulo BRAZIL
**Corresponding Author Secondary Information:**	
**Corresponding Author's Institution:**	Universidade Estadual de Campinas
**Corresponding Author's Secondary Institution:**	
**First Author:**	José Augusto Salim
**First Author Secondary Information:**	
**Order of Authors:**	José Augusto Salim
	Antonio M Saraiva
	Paula F Zermoglio
	Kayna Agostini
	Marina Wolowski
	Debora P Drucker
	Filipi M Soares
	Pedro J Bergamo
	Isabela G Varassin

Powered by Editorial Manager® and ProduXion Manager® from Aries Systems Corporation

	Leandro Freitas
	Márcia M Maués
	André R Rech
	Allan K Veiga
	Andre L Acosta
	Andréa C Araujo
	Anselmo Nogueira
	Betina Blochtein
	Breno M Freitas
	Bruno de Carvalho Albertini
	Camila Maia-Silva
	Carlos E P Nunes
	Carmen S S Pires
	Charles F dos Santos
	Eliza P Queiroz
	Etienne A Cartolano
	Flavízia F de Oliveira
	Felipe W Amorim
	Francisco E Fontúrbel
	Gleycon V da Silva
	Hélder Consolaro
	Isabel Alves-dos-Santos
	Isabel C Machado
	Juliana S Silva
	Kátia P Aleixo
	Luísa G Carvalheiro
	Márcia A Rocca
	Mardiore Pinheiro
	Michael Hrncir
	Nathália S Streher
	Patricia A Ferreira
	Patricia M C de Albuquerque
	Pietro K Maruyama
	Rafael C Borges
	Tereza C Giannini
	Vinícius L G Brito
**Order of Authors Secondary Information:**	
**Response to Reviewers:**	Reviewer #1:<br><br>- "Regarding this implementation I wonder if two key aspects are sufficiently highlighted (eg Fig 1): the interaction (a single interaction) has consequences (outcomes) for

BOTH partners. It seems the dwc:Event has a proper descriptor for the animal (dwc:ResourceRelationship class) but not for the plant: the obis:ExtendedMeasurementOrFact extension appears to deal just with characteristics of the plant, flower, nectar produced, etc, and not with the result or consequence of the interaction for the plant. So, I miss a dwc:ResourceRelationship for the plant (e.g., pollen.is.deposited, flower.damaged, nectar.robbed, etc.) as well as an obis:ExtendedMeasurementOrFact for the pollinator (e.g., morphological characteristics, phenology, etc.). I mean, the structure of relationships between boxes in Fig 1 should be symmetrical."

We changed the figure following the suggestion of the reviewer, and now the figure is symmetrical for plant and animal occurrences. We highlighted that the only on dwc:ResoureRelationship must be used since the interactions have a direction (the subject to the object), but any computational system or user is able to recovery the inverse interaction (e.g visitsFlowerOf -> hasFlowersVisitBy), so no additional dwc:ResourceRelationship is needed.

- "Obviously, depending on the amount of data collected or the specific objectives of the samplings, not all this information can be available. Yet the proposed vocabulary is detailed enough to capture most of the details of interest for any plant-pollinator interaction. I miss a brief comment about the minimal set of descriptors that could be considered to provide adequate information for this type of interaction, like a real world "example". That would be of interest to more general readers."

We added a paragraph about the minimum set of terms in order to provide adequate information about the interactions.

- "1. I'd suggest increasing font size in Fig 3 to improve readability."

The font size was increased. Now the Fig 3 is more readable.

Reviewer #2:

- "The authors emphasized that the interaction may be based on individual level. I agree with this. However, data on plant-pollinator interaction is not always collected at individual level. For example, a pollination network is often constructed based on pollen on pollinators. In such studies, it is impossible to know how many plant individuals have been visited by an individual pollinator. Moreover, a pollinator individual could carry pollen grains from multiple plant species. How can this type of data be treated as an interaction in this study?"

We added a brief section about how to document data in interaction networks ("Documenting plant-pollinator interaction networks"). We hope it clarify how networks can be standardized. It should not be different from the individual-level, since in networks the information is only structured in a different format, but essentially it is the same. Nodes can be documented as dwc:Occurrence and edges/links as dwc:ResourceRelationship, having the dwc:Event as link between dwc:Occurrences, dwc:ResourceRelationship and any measurement or facts which might exists.

- "Should pollinators be identified to species level in the data schema? I could not understand whether an interaction event in the data schema allows the interaction that a pollinator (or a plant) is not identified to species level. This is an important point because we can't always identify pollinators to species level. Moreover, we sometimes can't identify pollinator taxa even to family or genus level. For example, many studies on pollination have identified pollinators as not taxa rather than functional groups (e.g. "midge", "long-tongued bee" or "small beetle"). Does the data scheme allow interaction between a plant species and a pollinator functional group?"

We added a brief paragraph explain that DwC, and so, the schema presented in the manuscripts, does not make any assumption about the taxonomic rank level that should be given in the dwc:scientificName term. So, it is fine to use any level above (or bellow) species rank to document the organisms in the dwc:Occurrence. Theschema presented does not make any changes in the DwC scientificName term definition: "The full scientific name, with authorship and date information if known. When forming part

	of an Identification, this should be the name in lowest level taxonomic rank that can be determined." (https://dwc.tdwg.org/terms/#dwc:scientificName).<br><br>We would like to thank you the reviewers for the valuable comments and suggestions in order to improve the manuscript.<br><br>We hope that all the issues pointed by the reviewers have been addressed.<br><br>Sincerely,<br>José A. Salim
Additional Information:	
Question	Response
Are you submitting this manuscript to a special series or article collection?	No
**Experimental design and statistics**<br><br>Full details of the experimental design and statistical methods used should be given in the Methods section, as detailed in our Minimum Standards Reporting Checklist. Information essential to interpreting the data presented should be made available in the figure legends.<br><br>Have you included all the information requested in your manuscript?	Yes
**Resources**<br><br>A description of all resources used, including antibodies, cell lines, animals and software tools, with enough information to allow them to be uniquely identified, should be included in the Methods section. Authors are strongly encouraged to cite Research Resource Identifiers (RRIDs) for antibodies, model organisms and tools, where possible.<br><br>Have you included the information requested as detailed in our Minimum Standards Reporting Checklist?	Yes
**Availability of data and materials**	Yes

All datasets and code on which the conclusions of the paper rely must be either included in your submission or deposited in publicly available repositories (where available and ethically appropriate), referencing such data using a unique identifier in the references and in the "Availability of Data and Materials" section of your manuscript.<br><br>Have you have met the above requirement as detailed in our Minimum Standards Reporting Checklist?	

Powered by Editorial Manager® and ProduXion Manager® from Aries Systems Corporation

Click here to access/download;Manuscript;main.tex

OXFORD (GIGA)^n SCIENCE

GigaScience, 2017, 1–13

doi: xx.xxxx/xxxx
Manuscript in Preparation
Review

REVIEW

Data standardization of plant–pollinator interactions

José A Salim[1,*], Antonio M Saraiva[1, †], Paula F Zermoglio[31], Kayna Agostini[18], Marina Wolowski[14], Debora P Drucker[32], Filipi M Soares[1], Pedro J Bergamo[9], Isabela G Varassin[25], Leandro Freitas[9], Márcia M Maués[4], Andre R Rech[26], Allan K Veiga[1], Andre L Acosta[1], Andréa C Araujo[24], Anselmo Nogueira[21], Betina Blochtein[10], Breno M Freitas[22], Bruno C Albertini[1], Camila Maia-Silva[28], Carlos EP Nunes[30], Carmen SS Pires[5], Charles F dos Santos[10], Elisa P Queiroz[1], Etienne A Cartolano[1], Favízia F de Oliveira[15], Felipe W Amorim[11], Francisco E Fontúrbel[2], Gleycon V da Silva[7], Hélder Consolaro[15], Isabel Alves-dos-Santos[1], Isabel C Machado[17], Juliana S Silva[6], Kátia P Aleixo[3], Luísa G Carvalheiro[16], Márcia A Rocca[19], Mardiore Pinheiro[13], Michael Hrncir[1], Nathália S Streher[29], Patricia A Ferreira[12], Patricia MC de Albuquerque[23], Pietro K Maruyama[27], Rafael C Borges[8], Tereza C Giannini[8] and Vinícius LG Brito[20]

[1]Universidade de São Paulo ; [2]Pontificia Universidad Católica de Valparaíso ; [3]Associação Brasileira de Estudos das Abelhas ; [4]Embrapa Amazônia Oriental ; [5]Embrapa Genetic Resources and Biotechnology ; [6]Instituto Federal de Educação, Ciência e Tecnologia de Mato Grosso ; [7]Instituto Nacional de Pesquisas da Amazônia ; [8]Instituto Tecnológico Vale ; [9]Rio de Janeiro Botanical Garden ; [10]Pontifícia Universidade Católica do Rio Grande do Sul ; [11]Universidade Estadual Paulista "Júlio de Mesquita Filho" ; [12]Universidade Federal da Bahia ; [13]Universidade Federal da Fronteira Sul ; [14]Universidade Federal de Alfenas ; [15]Universidade Federal de Catalão ; [16]Universidade Federal de Goiás ; [17]Universidade Federal de Pernambuco ; [18]Universidade Federal de São Carlos ; [19]Universidade Federal de Sergipe ; [20]Universidade Federal de Uberlândia ; [21]Universidade Federal do ABC ; [22]Universidade Federal do Ceará ; [23]Universidade Federal do Maranhão ; [24]Universidade Federal do Mato Grosso do Sul ; [25]Universidade Federal do Paraná ; [26]Universidade Federal dos Vales do Jequitinhonha e Mucuri ; [27]Universidade Federal Minas Gerais ; [28]Universidade Federal Rural do Semi-Árido ; [29]University of Pittsburgh ; [30]University of Stirling ; [31]Universidad de Buenos Aires ; [31]Embrapa Agricultura Digital

*jasalim@unicamp.br
†saraiva@usp.br

Abstract

Background: animal pollination is an important ecosystem function and service, ensuring both the integrity of natural systems and human well-being. Although many knowledge shortfalls remain, some high-quality datasets on biological interactions are now available. The development and adoption of standards for biodiversity data and metadata has promoted great advances in biological data sharing and aggregation, supporting large scale studies and science-based public policies. However, these standards are currently not suitable to fully support interaction data sharing. **Results:** here we present a vocabulary of terms and a data model for sharing plant-pollinator interactions data based on the Darwin Core standard. The vocabulary introduces 48 new terms targeting several aspects of plant-pollinator interactions, and can be used to capture information from different approaches and scales. Additionally, we provide solutions for data serialization using RDF, XML and DwC-Archives and recommendations of existing controlled vocabularies for some of the terms. Our contribution supports open access to standardized data on plant-pollinator interactions. **Conclusion:** the adoption of the vocabulary would facilitate data sharing to support studies ranging from the spatial and temporal distribution of interactions, to the taxonomic, phenological, functional, and phylogenetic aspects of plant-pollinator interactions. We expect to fill data and knowledge gaps, thus further enabling scientific research on the ecology and evolution of plant-pollinator communities, biodiversity conservation, ecosystem services, and the development of public policies. The proposed data model is flexible and can be adapted for sharing other types of interactions data by developing discipline-specific vocabularies of terms.

Key words: biodiversity information; Darwin Core; vocabulary of terms; pollination; pollinator; biodiversity informatics

Background

Introduction

Pollination is a key natural process that provides indispensable ecosystem services and safeguards agricultural production and food security worldwide [1]. Almost 90% of flowering plant species [2], including more than half of the global crop species [3], rely to some degree on animal pollination for their reproduction [4, 5]. Concerned with the current global biodiversity crisis and its impacts on ecosystems and human health, the Convention on Biological Diversity [6] and the Intergovernmental Science-Policy Platform on Biodiversity and Ecosystem Services [7] acknowledged the importance of plant-pollinator interactions for ecosystem functioning and sustainable agriculture [8]. Although large datasets of plant-pollinator interactions data have become available worldwide, great challenges remain regarding data storage and standardization. These issues need to be solved to enable the development of integrative studies that allow attaining broad scale knowledge on species biology, phenology and evolution, as well as to support the decision making process for pollinator conservation. Before IPBES, many initiatives and funding programs were created to promote and support research and conservation of pollinators and plant-pollinator interactions adopting the concept of open data. Among the most prominent are the International Pollinators Initiative — The São Paulo Declaration on Pollinators [9], the Global Action on Pollination Services for Sustainable Agriculture of the Food and Agriculture Organization of the United Nations (FAO, fao [10]), the United States Geological Survey (USGS) Pollinators Conservation Program [11], and the European Union Pollinators Initiative [12]. Nevertheless, many data gaps still exist regarding plant-pollinator interactions (see, for example, Wolowski et al. [13], for an analysis of native species in the Atlantic forest). Initiatives like the IPBES have demanded quick access to high-quality spatial and temporal data of species occurrences, their interspecific relations, and the environmental effects on biotic interactions. These high-quality data have the potential to improve our knowledge about ecological and evolutionary processes guided by interspecific interactions, as well as to assist in planning and decision making for biodiversity conservation and restoration [14].

Primary data on pollinators is becoming increasingly available online and can be accessed from a large number of data repositories. Moreover, many initiatives have also been created to facilitate and to stimulate the dissemination of pollinators and plant-pollinator interactions data, such as the Inter-American Biodiversity Information Network - Pollinators Thematic Network (IABIN-PTN), the WebBee [15], the UK Plant-pollinator interactions database [16], and the Plant-Pollinator Interaction Explorer [17]. There are also more general initiatives that aim to organize data of all types of biotic interactions, for example, the projects Global Biotic Interactions - GloBI [18], Gulf of Mexico Species Interaction - GoMexSI [19], Mangal [20], Interaction Web DataBase (IWDB, Int [21], Kelpforest Database [22], the LifeWebs project [23], the GlobalWeb [24] and the Web of Life [25]. In spite of the increasing availability, there remain serious data gaps and biases. For instance, there is a larger amount of interaction data from temperate and high latitude regions compared to the tropics [26, 27], hampering the assessment of global patterns such as latitudinal gradients [28, 29]. Species interaction data, especially binary matrices or binary networks (i.e., presence/absence of interaction), can also be found in many scientific papers, but detailed information on each interaction and species traits is still sparse in the literature. Having scattered information has hindered answering urgent questions about the roles of species and their interactions within communities and ecosystems, and their impact on ecosystem functions and services [30, 31, 32], as well as understanding how pollinators behave or with whom they interact in different types of ecosystems or biomes.

Most currently available species-interaction datasets do not adopt any standard for data or metadata capture and annotation (e.g. Allen-Perkins et al. [33]). Moreover, for those that do, the lack of appropriate data standards largely contribute to the dispersion and heterogeneity in the data. Thus, data aggregation relies on laborious and repeated transformations of the original datasets into custom, non-standardized formats, making data integration and discovery a costly and time-consuming process. In addition, data on interactions recorded by different studies may impose limitations to the generalization of conclusions due to variation in sampling methods and research objectives, usually not documented in the metadata (e.g., Pimm et al. [34], Beas-Luna et al. [22]). As a result, species interaction data are often insufficient or biased for many types of analyses.

Broad-scale analyses require data to satisfy the FAIR principles (i.e., Findable, Accessible, Interoperable, and Reusable data, Wilkinson et al. [35]). Fulfilling such criteria is challenging, and it is essential that biodiversity standards (e.g., Findable, Accessible, Interoperable, and Reusable data) aid in meeting those principles. as they enable comparison of data from different contexts, shared through different open access global databases.

Findable, Accessible, Interoperable, and Reusable data (hereafter DwC, Wieczorek et al. [36]) is a standard for sharing data about life on Earth as documented by observations, specimens, samples, and related information. DwC was ratified as a standard in October 2009 by the Biodiversity Information Standards (TDWG) organization. Since then, it has been adopted by several communities around the globe. The most prominent case of DwC adoption is perhaps the Global Biodiversity Information Facility [37], which aggregates more than 1.9 billion biodiversity data records, as of January 2022. The DwC standard, as well as other standards for biodiversity data and metadata, such as Access to Biological Collection Data (ABCD [38], Audubon Core [39], and Ecological Metadata Language (EML, Jones et al. [40]), constitute a great advance in biological data sharing and aggregation, supporting the development of studies and science-based decision-making. However, a general, adaptive and comprehensive solution for biological interaction data standardization, including plant-pollinator interactions, is still not available.

Biological interactions usually include data that cannot be adequately represented by the DwC standard as it currently stands, because it lacks appropriate terms to document them in detail. Also lacking is a common model to express important components of the phenomena, such as the type, direction, effects and outcomes of an interaction. However, DwC is flexible enough to be extended and new terms and controlled vocabularies may be created to accommodate new use cases. For example, DwC has been extended to support standardization of genomic data [41, 42] and zooarchaeological data [43], and several other extensions are currently being used or in development by the community [44]. The latest version of DwC (version 2021-07-15) also incorporates four controlled vocabularies of values.

Every (pairwise) interaction involves two organisms or two groups of taxonomically homogeneous organisms which per-

Compiled on: March 29, 2022.
Draft manuscript prepared by the author.

form a co-action at a particular place and time [45]. While the taxonomic, spatial, and temporal information about the occurrences of such organisms or group of organisms can be documented using DwC, there is no formal or recommended process to express the association of such occurrences, and the particularities of an interaction.

Despite that, biological interactions data have being documented using many different approaches, including the adoption of the "Association terms" from the DwC standard (i.e. `dwc:associatedTaxa` and `dwc:associatedOccurrences`) and the `dwc:ResourceRelationship` class. We also find some non-conventional ways to document interactions using the terms `dwc:occurrenceRemarks` and `dwc:dynamicProperties` and those in the class `dwc:MeasurementOrFact` (MoF). There is also a non-standard DwC "association extension" [46] developed by Encyclopedia of Life (EOL) which focuses primarily on taxonomic characteristics of the interactions, instead of their ecological and functional aspects.

To extend our capacity to share interactions data, in this paper we present a vocabulary of terms to document plant-pollinator interactions developed by a community of specialists, and a data model to use the vocabulary based on DwC. The remainder of the paper is organized as follows: first, we provide an overview of the previous initiatives regarding plant-pollinator data, which have paved the way for this work. We then introduce and discuss the process of community driven vocabulary development, and present the plant-pollinator interactions vocabulary itself. Lastly, we present the use of DwC to document biological interactions, including the plant-pollinator interaction data model for representations using DwC-Archives, XML, and RDF, and draw some conclusions.

Historical overview on plant-pollinator interactions data standardization

The plant-pollinator interactions vocabulary presented here started to be assembled in 2006, based on the demand from the Inter-American Biodiversity Information Network project, named Pollinators Thematic Network (IABIN-PTN). The initiative aimed at digitizing pollinator data for the Americas, including information on species occurrences, usually provided by biological collections and museums, as well as ecological relevant information on plant-pollinator interactions that were at the time seldom digitized [47]. In a joint effort with the FAO, a first solution proposed the use of three extensions to the existing Findable, Accessible, Interoperable, and Reusable data (named DwC v1.4, not yet a TDWG standard). They consisted of 1) a generic Interaction Extension intended to represent any observed interaction between two individuals, not restricted to pollinators or plants; 2) a Pollination Extension including additional data specific to plant-pollinator interactions, for instance pollen or nectar removal; and 3) an Environmental Measurement Extension to include the environmental conditions during the observation or collecting event. That proposal was published on the Darwin Core wiki [48] for broader discussion within the TDWG community, and attracted some attention. Despite the benefits of being a more generic approach, the discussions showed that it would require a lot of effort to reach consensus and, given the time constraints of the project, it was decided to focus only on plant-pollinator interactions. The subsequent version of the so-called Interaction Schema treated each interaction record as a triad: one DwC record for each of the two interacting organisms, and an interaction record that referenced the individual DwC records by means of their globally unique identifiers. The interaction record also included data about the type of interaction (e.g., collecting pollen, collecting nectar), observer, location, and date/time of the interaction. This approach allowed multiple interaction records to be associated with the same interacting individual [49].

Further simplification led to adopting only two terms to characterize the interaction: one field for the type of interaction, and another field for remarks, typically used for a bibliographic reference of the interaction. That was, finally, the solution adopted for the system and tools developed during the IABIN-PTN project [50]. The same approach was used for digitization of interaction data collected within the GEF/UNEP/FAO Global Pollination Project on the "Conservation and Management of Pollinators for Sustainable Agriculture, through an Ecosystem Approach", which involved partners in seven countries: Brazil, Ghana, India, Kenya, Nepal, Pakistan and South Africa [51]. Overall, those projects have enabled the digitization of thousands of plant-pollinator records that follow the same template and can, thus, be reused more easily.

Knowing the potential and importance of adding richer data content to each interaction, another attempt was made to further evolve the pollinator interaction data standard. Also supported by FAO, a survey of potential descriptors of plant-pollinator interactions was conducted with researchers from five continents [51]. The 23 participants shared the data fields they used to digitize interaction data for their research purposes. As their research questions varied, so did the fields they used in their spreadsheets (most cases) or databases (a few cases). The result of that compilation was a very long list of data fields (more than two hundred), which included terms related to the plant (taxonomy, traits), the potential pollinator or flower visitor (taxonomy, traits), the experimental setting and protocol, the environment, the outcome of the interaction (such as fruit set), and references, among others. As for the interaction-related fields, some referred to pollinators behavior, some to resources collected, to interaction frequency, results or outcomes. Many of the descriptors suggested by different authors seemed to be synonyms (as one might expect), but they were not accompanied by a clear description of their meaning and form of usage (semantics and syntax), which made it difficult to compare and coalesce them. That list was clearly very valuable, but needed a detailed evaluation and intense work to compare, categorize, sort, and define the fields so as to identify a good set of candidate terms. That required experts in the fields involved - pollination ecology, botany, zoology, information and computing science, to create a community-driven vocabulary, which is critical for the development of a proposal that really reflects the vision and the needs of a broad community, a prerequisite for a data standard [52], and fosters its subsequent adoption.

With its founding members aware of the previous context, the Brazilian Network of Plant-Pollinator Interactions (REBIPP) was established in 2016 with the aim of encouraging scientific, educational and outreach activities related to Pollination Biology. REBIPP is a collaborative network of specialists in Pollination Biology, researchers of plant-pollinator interactions in its various scales and dimensions, and one of its original objectives was the development of a Brazilian Plant-Pollinator Interaction Database. Having to deal with the current standards shortcomings firsthand, and increasingly involved, members of the network were keen to take a step further into developing a more comprehensive solution. Building on the momentum of this community engagement, REBIPP seized the opportunity to broaden its original objectives, welcoming other members of the international community, to jointly develop a solution for sharing standardized plant-pollinator interactions data.

Community-driven vocabulary development

During 2017 and 2018, specialists on pollination biology and information science from the Brazilian and Chilean networks

on plant-pollinator interactions met in four workshops to review and discuss those descriptors of plant-pollinator interactions defined in the previous initiatives mentioned above. The meetings aimed to engage all participants in discussions and to reach consensus about the terms that would compose the standardized vocabulary for plant-pollinator interaction data. Specialists worked simultaneously in three task groups (plant, animal and interaction group), each focused on the revision and definition of specific terms. The first two groups focused on terms to describe relevant plant or animal traits, while the interaction task group reviewed the descriptors that characterize the interactions. In order to facilitate reconciling the vocabularies from different groups, a common template was used (Table 1). Periodically, the task groups engaged in all-hands discussions, so that each group could get acquainted with the progress and decisions made by the others.

Reaching a consensus on terms and their definitions among members of each group was challenging, since researchers have different views and concerns about which data are important and must be represented in a standard, as expected in any scientific field (e.g., Tremblay et al. [53]. Thus, we ended up having a list of 278 terms, many of which were useful only in very specialized research protocols and experiments, rarely collected or measured. Following the metadata principle of simplicity [54, 55, 56], on the premise that a standard with too many terms is difficult to use and complexity imposes barriers to its adoption, we started a second round of terms review. For this, the task groups worked together on the whole set of terms to refine the definitions and reduce the list to a core of important concepts related to plant-pollinator interactions. With a reduced list of 278 terms, we built a draft version of the vocabulary, and specialists in biodiversity, informatics and information science worked together to validate and refine it, ensuring it would be compliant with current standard wording and practices. Finally, we performed a "Community Review" among all participant members to solve any conceptual and practical problems and validate the vocabulary using real data examples. To conduct the review we used GitHub Issues Tracking [57], which was essential for the process to be transparent and open access, and the template to formalize the definitions of terms, which also helped the organization of the vocabulary.

After almost three years of collaborative and voluntary work, the first version of the plant-pollinator standard was concluded. The vocabulary includes 48 new terms specifically defined for plant-pollinator interactions (see Supplementary Material), which can be accessed through the open access and stable repository [58]. Additionally, we provide controlled vocabularies for many terms which bring the definition of new CV terms or import terms from other existing vocabularies [59]. It is important to note that the terms discarded from this first release of the vocabulary and their history have been kept in the GitHub repository and can be revisited in the future as the vocabulary evolves.

Guidelines to collaborative creation of a new vocabulary of terms

The collaborative creation of a vocabulary involves many challenges with different levels of complexity. This complexity is partially related to the empirical and sociological components of a collaborative and democratic community. In order to facilitate and help other biodiversity information communities in the creation of their own standards and vocabularies, we elaborated a set of guidelines covering aspects from the conceptualization to the adoption of a vocabulary. The guidelines were elaborated mainly based on our experience during the creation of the plant-pollinator interaction vocabulary. Although the technical details of how to build a standard are already documented in the TDWG Vocabulary Maintenance Standard (VMS, Group [60]) and in the TDWG Standards Documentation Standard (SDS, Group [61]), there is no guidance on how a community should organize itself and how members should collaborate to democratically reach an agreement. For that reason, here we propose a workflow which can be used and adjusted by other communities according to their needs and requirements.

The workflow is summarized in the following steps (1):

- **Identification of key stakeholders:** this is probably the most critical step. Engagement from the community is vital not only for the development of a vocabulary but, more importantly, for its later adoption. It is important for the members of the community to clearly understand the benefits of data standardization and also to give them a sense of ownership over the resulting products. However, there should be a careful balance between representativeness and group size. The formation of smaller and more homogeneous working groups focusing on specific parts of the vocabulary being created should follow some predefined criteria. This promotes having more operative and efficient groups. In our case, the community was divided in three groups according to each member's expertise in botany, zoology and ecology. The size of the groups is dependent on the size of the whole community, but working groups that are unbalanced in size may lead to biased perceptions and definitions of the main topic.
- **A formal definition of the main topic:** the main topic (including main goals and challenges) must be defined early in the creation of the vocabulary. Conceptualizing the main topic may involve the abstraction of complex and sometimes ambiguous concepts (in the case of pollination ecology, e.g., "species", "specimen", "traits", "functional traits", "legitimate pollinator", "floral visitor"), thus it is essential to have a robust and formal definition of the subject matter. Expert knowledge may facilitate the definition of the main topic, but it should not be the only source of knowledge (e.g., literature, glossaries, nomenclature codes should also aid the process). The formal definition needs to be based on an agreement reached by the community to set a clear scope and avoid ambiguities and conflicting concepts. The definition may be created by borrowing concepts and terms from other standards and vocabularies.
- **Collaborative compilation of an initial set of terms:** to describe the data domain (what we refer to as "variables" or "descriptors" to avoid confusion with a formal term which will be part of the standard or the vocabulary). This may include assessment of terms from existing standards. When reusing terms from existing standards and vocabularies, attention should be given to avoid misunderstanding of the concepts represented by the terms. For that reason, this step should only focus on the description of the concepts ("descriptors") instead of their representation as a formal vocabulary term (i.e., an entity representing a concept) [60]. The working groups should collaborate to compile an inclusive set of descriptors that contemplates a general understanding of the concepts, but conflicting concepts may be accepted depending on the heterogeneity of the whole community. In more homogeneous communities where members already have a consensus about the concepts to be represented by the vocabulary, the following steps and the creation of the vocabulary may be simplified.
- **Review and refinement:** the initial set of descriptors provides a preliminary definition for the terms. Those terms should be subjected to rounds of review to refine their definitions, examples of usage and to elaborate glossaries for controlled vocabularies (if applicable). This is the step in

Table 1. Template used to define the terms in the plant-pollinator interactions vocabulary. *Term Label*: a human readable name; *Identifier*: a unique Internationalized Resource Identifier (IRI) for the term in namespace; *Class*: the category in which a term is defined. It is not a formal class definition (aka `rdf:Class`); *Definition*: term definition in a human readable form; *Comments*: any additional comments to the term definition and its use; *Details*: a reference for the concept represented by a term; *Protocol*: recommended protocols to measure or to record the value for the term (if applicable); *Controlled Vocabulary*: list of recommended values, such as terms from existing thesauri or ontologies (if applicable).

**Term Label: Flower Opening Type**	
**Identifier**	http://rs.rebipp.org.br/ppi/terms/flowerOpeningType
**Class**	Flower
**Definition**	The type of flower describing whether the flower's corolla opens or not, exposing its reproductive parts
**Comments**	Recommended best practice is to use a controlled vocabulary.
**Details**	Proctor, M. P. et al. 1996. The natural history of pollination. HarperCollins. Inouye DW, Favre DW, Lanum JA, Levine DM, Meyers JB, Roberts MS, Tsao FC, Wang Y-Y. 1980. The effects of nonsugar nectar constituents on estimates of nectar energy content. Ecology 61: 992–996
**Protocol**	Observation of the floral development from the bud stage to senescence (Dafni et al. 2005).
**Controlled Vocabulary**	cleistogamous; chasmogamous; both
**Examples**	cleistogamous; chasmogamous; both

which terms can be merged if their definitions reflect the same concept, or split into two or more terms if a need is identified. If the set of terms includes a large number of conflicts it can be split into multiple more specific sets (which may lead to multiple vocabularies). Alternatively, the community may try to deal with conflicting terms by defining more general concepts. Terms can also be excluded. We do not recommend adding new descriptors at this step (unless by splitting existing descriptors or by adopting terms from existing vocabularies) because the addition of new terms would result in unnecessary vocabulary growth and a larger number of review rounds to reach a consensus. The working groups should focus on the review and refinement of the descriptors related to their objectives and use cases, but they may collaborate with each other, especially to adopt terms from existing data standards. New terms should only be defined if they are not already defined by other data standards. The process described in this step should be iterated until full consensus is reached.

- **Minting new terms:** the refined set of terms should be mapped to other community vocabularies (e.g., DwC), as should the recommended controlled vocabularies (if applicable). Sometimes a term can be similar to a term already defined in another available vocabulary, but with slightly different semantics or formatting. Whenever possible, it is recommended to review the new term to match an existing term definition. If this is not feasible or appropriate, the new term should be created and added to the draft version of the vocabulary. It is also possible to propose a change to an existing term in other standards.
- **Elaborate a representation model:** it is also advisable to elaborate at least one representation model for the vocabulary and document its usage under different schemas. Our recommendation is to at least declare the terms using RDF to improve the interoperability with other communities, but also to provide other schemas if possible, such as Darwin Core Archives, and XML. It is important to consider solutions already adopted by a broader community in order to maintain the interoperability and consistency among models and schemas. If the data model is too complex or the vocabulary can not be represented in one of the chosen schemas, go back to step 4 and try to refine the terms in order to reduce any dependencies among the terms (e.g., a term for which the interpretation depends on the value of another term, a multilevel of many-to-one or many-to-many relationships between the terms).
- **Validation:** compiling a set of real data covering different scientific questions and verifying if the vocabulary can capture all or the most relevant information needed for each use case. If any issue is detected (e.g., missing or conflicting definitions) or there is any ambiguity in the definition of the terms, go back to step 4 and refine the set of terms to overcome it.
- **Make the standard broadly available:** (e.g., through formal publication or collaborative platforms) so that members of other communities can openly access it for use and be involved in its evolution. For this purpose, GitHub [62] has proven to be a useful platform for tracking and maintaining standards. However, keep in mind that other layouts may be needed for those audiences that are not familiar or comfortable with GitHub repositories. Creating a simple, friendly web page with a description of the vocabulary and its purpose and where users can browse for terms and definitions may be considered (a model for this is the DwC Quick Reference Guide [63]).

Data Quality and Controlled Vocabularies

The adoption of controlled vocabularies contributes significantly to data quality and interoperability of biodiversity datasets from the same and other communities, as it makes data easier to find and use [64]. Although the plant-pollinator interactions vocabulary does not restrict how data is captured under each term, it provides some guidelines for the adoption of thesauri and ontologies, when available. . There are many ontologies available that can enrich data description and annotation and that are relevant to plant-pollinator interactions data, such as the Plant and the Plant Trait Ontologies [65], the Phenotype and Trait Ontology [66], the Flora Phenotype Ontology [67], the Hymenoptera Anatomy Ontology [68], the Vertebrate Ontology [69] and Uberon [70]. Moreover, ontologies are also available for environment description, like the Environment Ontology [71], and biotic interactions, such as the Relations Ontology[72]. Recommendations to use particular controlled vocabularies are not meant to be normative and, since there are values that can not be mapped to any existing controlled vocabularies, we provide some for specific terms. As new or revised vocabularies emerge from the community, such recommendations should be easily amended, accompanying and facilitating the evolution of the vocabulary.

A plant-pollinator interactions vocabulary

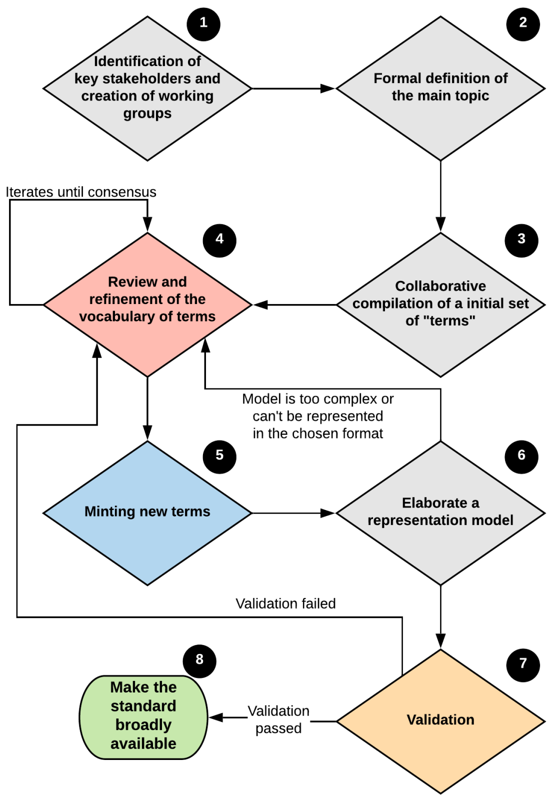

Figure 1. Suggestion of a community workflow to create a new vocabulary of terms. Numbers in the black circles indicate the steps described above in the text.

Defining an Interaction

The potential uses of the broad and heterogeneous term "interaction data" need to be circumscribed by providing a common definition and understanding shared by members of different communities. Achieving an agreement in the definition of an "interaction" in its broadest context (e.g., "behavioral interaction", "ecological interaction") proved to be challenging, as expected provided the long-standing debate around the subject in the ecological literature [73]. Even when restricted to plant-pollinator interactions, the diversity of background knowledge and expertise in a multidisciplinary field such as pollination biology resulted in divergent and conflicting concepts of what an "interaction" is according to different perspectives (e.g., plant-centered perspective, animal-centered perspective, interaction among species, interaction among individuals).

Those conflicting concepts lead to an initial proposition of a great number of variables to describe and characterize an "interaction". In addition, the high level of abstraction of these concepts requires a subjective causal inference that is beyond what primary data can represent. Unlike recording the occurrence of an organism, which is restricted to spatial and temporal scales, recording an "interaction" also involves a subjective human interpretation about the biological meaning and effects of such interaction. There are also several contingencies (e.g., for competitive exclusion, Pedruski et al. [74]) and multiple definitions being adopted (e.g., for mutualism, Mazancourt et al. [75], and symbiosis, Martin and Schwab [76]). In spite of discrepancies, there is a consensus in the community that an "interaction" is a context-dependent action that occurs at a particular location during some time. By "context-dependent" we mean that interactions are dependent on the habitat and the species- or individual-level traits of the interacting organisms [77], as well as on the presence/absence of individuals of other species (e.g., higher-order interaction, Werner [78]).

Another widely discussed topic is related to the level at which interactions are recorded. While some members of the community emphasize that an interaction should be documented at the species-level, others argue that it should be documented at the individual-level [79]. Species-level interactions have been historically documented, especially in community ecology, yet they consist of a summary of interactions that are actually recorded in the field at the individual-level [80]. Since DwC is "primarily based on taxa, and their occurrence in nature as documented by observations, specimens, samples, and related information", it makes sense to document interactions at the individual-level. Additionally, species-level interactions could be derived from aggregating individual-level interactions, but not in the opposite direction. Thus, we adopted the following definition of an interaction:

> **Interaction:** a context-dependent action that a particular Organism or group of organisms (considered to be taxonomically homogeneous) perform on another particular Organism or group of organisms (taxonomically homogeneous) living together in a community at a particular location during some time.

Where an Organism is defined as: "*A material entity that is an individual living system, such as animal, plant, bacteria or virus, that is capable of replicating or reproducing, growth and maintenance in the right environment. An organism may be unicellular or made up, like humans, of many billions of cells divided into specialized tissues and organs*" [81].

Our definition emphasizes the individual perspective of the interactions in contrast to the species perspective [80]. Additionally to the spatial and temporal elements of an interaction, there are several context-dependent characteristics which are important to further interpret any particular interaction. According to Jordano [82, 83] any interaction is composed of three basic components: the co-occurrence, the encounter, and the outcome. To allow more efficient data aggregation and analysis, these three components should be properly documented. With that in mind, we propose a vocabulary that includes terms to capture specific details about these components, documenting the different contexts in which the interactions take place.

Although the interactions being documented at the individual level, it does not require that the interacting organisms must be taxonomically identified at the species rank level. Following the DwC standard, organisms can be identified at any taxonomic rank (usually the lowest level taxonomic rank that can be determined). It is important since some studies do not identified the plant or pollinators at the species taxonomic rank. Some studies on pollination have identified organisms as functional groups, despite the DwC standard does not provide a specific term for documenting functional groups, it can be achieved by using the *remarks* terms in DwC (e.g `dwc:taxonRamarks`, `dwc:occurrenceRemarks`) and providing the lowest taxonomic rank in the `dwc:scientificName`.

The vocabulary

The plant-pollinator interactions vocabulary comprises a set of documents, including both machine- and human-readable forms. The vocabulary is composed of a list of terms, their definitions, usage comments and examples, as well as a set of descriptive documents explaining how to use the vocabulary [58]).

The terms in the vocabulary are divided into six categories: Animal, Plant, Flower, Interaction, Reproductive Success, and Nectar Dynamics. The categories are not formally defined

as classes and they are not part of the vocabulary, they are used only to organize the terms and to facilitate understanding by humans. The vocabulary includes a Term List (aka `tdwgutility:TermList`) with definitions of 48 terms represented as `rdf:Property`.

Each term in the vocabulary contains the following normative elements as defined in the TDWG SDS:

- **Term name:** a controlled value that represents the property or concept described by the term definition;
- **Term label:** a word or short phrase that serves as a human-readable name for the term;
- **Term IRI:** the HTTP IRI that uniquely identifies the current term;
- **Definition:** the normative definition of the term, written in English;
- **Modified:** the date in ISO 8601 Date format on which the most recent version of the term was issued;
- **Type:** the type of term (can be "Class", "Property", or "Concept"), in our case all take the value "Property".

Additionally, for each term we provide usage comments and examples as non-normative content.

Using Darwin Core to document biological interactions

The biodiversity community has found many different approaches to partially overcome the limitations of Darwin Core to share interactions data. Here we discuss the most common approaches presenting their advantages and disadvantages.

The "Association terms"

The DwC standard defines terms that can be used to document generic associations between instances of the *dwc:Occurrence* class and other resources (images, references, taxa, occurrences), which indicate that a resource was linked to a related resource of some type. Those terms are known as "Association terms" and include terms to document associations with taxa (`dwc:associatedTaxa`) and occurrences (`dwc:associatedOccurrences`). While the `dwc:associatedTaxa` term is meant to express an association of any kind (not only biological interactions) between an `dwc:Occurrence` and names of taxa, the term `dwc:associatedOccurrences` is meant to express an association between an `dwc:Occurrence` and one or more other `dwc:Occurrence`'s. The term `wc:associatedTaxa` is defined as "*A list (concatenated and separated) of identifiers or names of taxa and the associations of this Occurrence to each of them*" (example: "pollinator of:" "*Fuchsia magellanica*"). However, the usage of this term is not well established, and we can find values with many different patterns in the available data (see Supplementary Material for a list of associations extracted from datasets in the GBIF registry). This approach is limited to documenting the taxonomic component of an interaction, and does not allow capturing other interactions-related data or organism traits. Additionally, the nature of the association is often unspecified or, when documented, it does not adopt any controlled vocabulary, which makes data less reusable and difficult to aggregate. In turn, using the term `dwc:associatedOccurrences` conveys similar problems, and therefore none of them are adequate to document biological interactions, since both lack many important data elements to correctly contextualize biological interactions (e.g., spatial and temporal information, interaction outcomes).

Using `dwc:associatedTaxa` is useful, for example, when one wants to document plant occurrences and the names of taxa visiting those particular plants, without documenting the individual visitors. However, due to the definition of the term `dwc:associatedTaxa`, we cannot assume that the association reported was observed as part of the same interaction event (e.g., the term may include one or more associations recorded at a previous time). A similar problem arises when using the `dwc:associatedOccurrence` term: we cannot assume that the location and the time documented for both associated occurrences are the same as those of the interaction, no matter how obvious it might be, since the definitions of the terms are too generic to support that assertion.

The `dwc:ResourceRelationship` class

The DwC standard also provides a solution to document any kind of relationship between records (e.g., occurrences, taxa, locations, identifications) in a more detailed way compared to the "Association terms". The current version of DwC includes a comment in the description of the terms `dwc:associatedTaxa` and `dwc:associatedOccurrences` which recommends the usage to `dwc:ResourceRelationship` class as an alternative to representing associations in more detail. Because of that the `dwc:ResourceRelationship` class has been adopted by some initiatives and previous studies to document biological interactions [84, 85]. Although it allows to document biological interactions in more detail, there are some limitations to document characteristics of the relationships themselves (e.g., interaction outcomes).

Currently, DwC does not provide a solution for `dwc:ResourceRelationship` class using RDF, due to the way in which DwC "ID" terms are defined. Darwin Core contains a number of "ID" terms intended to designate identifiers (e.g., `dwc:occurrenceID`, `dwc:identificationID`, `dwc:locationID`). The "ID" terms observe two functions, specifying the class of the resource and indicating that the value of the term is an identifier. However, in RDF, these two functions are handled separately using `rdf:type` declarations and URI's for expressing the identifier of the subject resource. For most DwC "ID" terms, the Dublin Core `dcterms:identifier` can be used as a replacement to indicate the identifier of an RDF resource, but the same cannot be applied to `dwc:ResourceRelationship`. The `dwc:ResourceRelationship` class includes two "ID" terms: `dwc:resourceID` ("*An identifier for the resource that is the subject of the relationship*"), and `dwc:relatedResourceID` ("*An identifier for a related resource; the object, rather than the subject of the relationship*"). Because the definition of `dcterms:identifier` makes a clear assumption of its usage ("*an unambiguous reference to the resource within a given context*"), it is not clear whether `dwc:ResourceRelationship` would make sense in the context of RDF, as a `dcterms:identifier` would make no distinction between each term it serves as a replacement for (i.e., `dwc:resourceID` or `dwc:relatedResourceID`). The latest version of DwC includes the new ID term (`dwc:relationshipOfResourceID`) which has reactivated the discussion about representing the `dwc:ResourceRelationship` in RDF, but it is still under debate and beyond the scope of this paper (see Baskauf and Webb [86] and New [87] for a discussion on this topic).

The "RDF world" is in its early stages of adoption by the biodiversity community [86]. However, there are some examples of `dwc:ResourceRelationship` usage to document biological interactions, as in the Catalogue of the Rust Fungi of Belgium [88], and in Plinian Core [85]. As the use of RDF grows, the RDF representation of biological interactions can be revised to properly meet the requirements of the Linked Open Data principles.

It is relevant to mention that the adoption of `dwc:ResourceRelationship` as a solution for documenting and sharing species interactions by previous initiatives does

not include the development and use of a common vocabulary. Instead, the dwc:ResourceRelationship is used only to link occurrences, taxa and specimens, but not to provide any data concerning the interaction itself (e.g., spatial and temporal information), nor additional information related to the interaction.

Non-conventional approaches

There are also other terms which have been used to document biological interactions, like dwc:occurrenceRemarks (e.g., Scheinberg [89]), and dwc:dynamicProperties (e.g., Cheadle Center for Biodiversity and Ecological Restoration [90]). The problem is that these generic terms expect either free text content (e.g., dwc:occurrenceRemarks) or content to be captured in some format that is cumbersome for both the data publishers and users (e.g., dwc:dynamicProperties recommends formatting data using key:value encoding schemas).

The DwC standard does not include atomized terms for capturing interactions-related data or organisms traits, but it includes generic terms for documenting additional measurements, facts, characteristics, or assertions about a record (e.g., dwc:measurementType and dwc:measurementValue, included in the dwc:MeasurementOrFact class). Although less common than other approaches we find examples of adoption of the dwc:MeasurementOrFact class to document biological interactions (e.g., [91]). Since the DwC standard itself does not contain controlled vocabularies of values for these terms, different communities have instead come up with some discipline-specific vocabularies, which are often not equivalent and difficult to compare to each other.

The plant-pollinator interaction data model

The main concern with the previous approaches is that the interactions are not the central piece of information being documented, instead they are treated as a link between occurrences or names of taxa. Since we are attempting to document interaction records between groups of organisms, the most appropriate approach is to represent any interaction as an instance of dwc:Event class. This approach is very similar to the one already being used in the GBIF network to document "sampling-event data" [92]. The main difference here is that, instead of sampling occurrences, we are documenting interactions between organisms, and their occurrences.

In DwC, the dwc:Event class is defined as "*an action that occurs at some location during some time*" [93], which is particularly generic and encompasses the definitions of "intra-action" and "co-action" suggested by Lidicker [45]. Thus, these definitions support the adoption of the dwc:Event class to represent a biological interaction, and in the plant-pollinator interaction data model the dwc:Event class is used to represent temporal and spatial details about the interactions. Therefore, a dwc:Event is linked to instances of dwc:Occurrence class representing the occurrences of the interacting organisms. In order to express the type and direction of the interactions different approaches are taken depending on the implemented application schemas (DwC-Archive, XML or RDF). Additionally, the dwc:MeasurementOrFact class should be used to represent any other characteristics of the interactions (e.g., outcomes) or occurrences (e.g., organism traits). The terms in the plant-pollinator interactions vocabulary should be used as values for dwc:measurementType. For those terms in the vocabulary that recommend the use of a controlled vocabulary the dwc:measurementValue can be used referencing the appropriate controlled values.

Like in DwC, none of the terms are mandatory. Ideally the more information provided the better. However, depending on the amount of data collected or the specific objectives of the studies, only a portion of the terms can be filled. In order to make interaction data reusable in different contexts there is a minimal set of terms which must be used. Thus, every interaction must provide, at least, following details:

- dwc:Occurrence: at least the taxonomic information must be present (dwc:scientificName).
- dwc:Event: at least the identifier of the event (dwc:eventID).
- dwc:ResourceRelationship: providing values for dwc:resourceID (the subject of the interaction), dwc:relatedResourceID (the object of the interaction), dwc:relantionshipOfResource or dwc:relantionshipOfResourceID (the interaction type; it can be very broadly defined such as *ecologically related to* or *participates in a biotic-biotic interaction with*) and for dwc:relationshipAccordingTo (the source of the interaction: person, organization, publication or reference).

Plant-pollinator interactions data as Darwin Core-Archive

Although documenting species interactions in text files and XML formats is less restrictive, the DwC-Archive [94] can be used to represent relations between resources (e.g., occurrence, taxon). While XML and RDF serialization formats can naturally handle one-to-many relations, the dwc:ResourceRelationship class, and also the dwc:MeasurementOrFact class, are of very limited use within Simple Darwin Core, as one-to-many relationships cannot be represented in "flat" files. The solution adopted by the DwC-Archive is to use a star schema, where a "core table" is linked to many "extension tables" by means of a unique identifier assigned to each record in the core table (i.e., the core id). Thus, it is possible to have multiple records in the extension tables referencing one record in the core table (i.e., implementing a one-to-many relationship).

For the scope of plant-pollinator interaction data we have extended the well known "sampling event data model". In the extended model, similar to the original model, the "core table" in the DwC-Archive represents events (instances of dwc:Event class), and an "extension table" is used to record the occurrences (instances of dwc:Occurrence class) related to each event in the core table. The difference is that, in the extended model, multiple characteristics of each event should be documented using either the dwc:MeasurementOrFact class or obis:ExtendedMeasurementOrFact (eMoF) extension [95].

The MoF class can be used to express one-to-many relationships of features associated with the target events (interactions) represented with the dwc:Event class, i.e., depicting the characteristics of the interactions. However, it cannot be used directly to document a characteristic of a plant or an animal participating in a particular interaction when DwC-Archives are used to standardize data, due to limitations of the star-schema. Although some workarounds have been attempted to overcome this, they are cumbersome both for data providers and users.

Otherwise, the eMoF extension was specially designed to handle environmental data in conjunction with species occurrence data. The eMoF extension is built on the existing dwc:MeasurementOrFact, using dwc:occurrenceID and adding three new terms: obis:measurementTypeID, obis:measurementValueID and obis:measurementUnitID. The dwc:occurrenceID term is used to circumvent the limitations of the star schema, and link measurement records in the obis:ExtendedMeasurementOrFact extension to occurrence records in the dwc:Occurrence extension (Figure 2). The other three terms are used to constrain and standardize the measure-

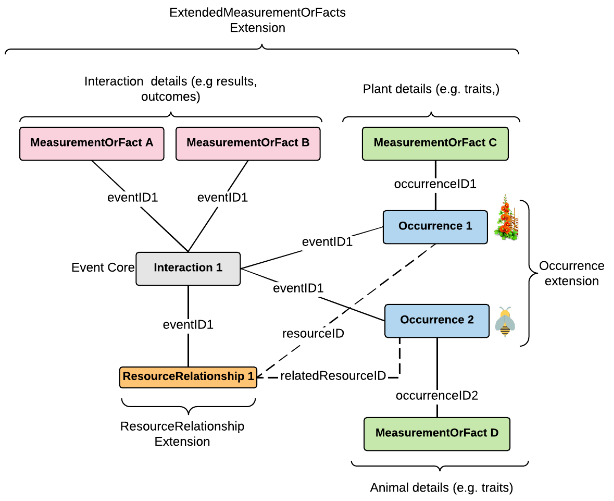

Figure 2. Overview of the data schema to represent plant-pollinator interactions. The plant and animal occurrences (blue boxes) are linked to the interaction (`dwc:Event`, grey box) using the `dwc:eventID` (full lines). Measurements related to the interactions (`MeasurementOrFact A` and `MeasurementOrFact B`, pink box, e.g., `ppi:resourceCollected`, `ppi:nectarCollectingBodyPart`, `ppi:numberOfRemovedPollenGrains`) are linked directly to the interaction (grey box) using the `dwc:eventID`. Measurements related to the occurrences (`MeasurementOrFact C` and `MeasurementOrFact D`, green boxes, e.g., `ppi:flowerColor`, `ppi:floralAttractants`, `ppi:caste`) are linked to the interactions using the `dwc:eventID` and the `dwc:occurrenceID` fields of the `obis:ExtendedMeasurementOrFact` extension (dashed lines). The direction and the type of the interaction are given by the `dwc:ResourceRelationship` class (orange box), linked directly to the interaction using `dwc:eventID` (full lines) and indirectly to the occurrences using `dwc:resourceID` and `dwc:relatedResourceID` terms (dashed lines)

.

ment types, values and units, since the `dwc:MeasurementOrFact` terms are completely unconstrained and can be populated with free text content. As stated by the authors: "*the three new terms should be populated using controlled vocabularies referenced using URIs)*" [95].

Thus, when using eMoF, the terms in the plant-pollinator interactions vocabulary should be used as values for `obis:measurementTypeID` and the `obis:measurementValueID` can be used referencing terms in controlled vocabularies providing the appropriate URI. The classic `dwc:measurementType` and `dwc:measurementValue` should be used to capture human-readable representations of the values used in the corresponding ID fields.

Additionally, in the extended model, the `dwc:ResourceRelationship` class is used to document the relationships between the occurrences. The `dwc:ResourceRelationship` class allows to document the type of the interaction (`dwc:relationshipOfResource` and `dwc:relationshipOfResourceID`) and the direction of the interaction, since the `dwc:ResourceRelationship` has terms for the subject (`dwc:resourceID`) and object (`dwc:relatedResourceID`) of the relationship (`dwc:resourceRelationshipID`). For the type of the interaction it is recommended to use values from the Relations Ontology [72]. The Plant-Pollinator Interactions vocabulary includes a guide explain how to document plant-pollinator interactions using DwC-Archive schema [96].

Plant-pollinator interactions data as XML

The implementation using XML is very similar to the implementation using DwC-Archive. The main difference is that in XML we don't have the limitations of the star-schema, and one-to-many relationships can be handled naturally. When using XML we don't need to use `obis:ExtendedMeasurementOrFact` to document additional characteristics of the `dwc:Occurrences`. Instead, the `dwc:MeasurementOrFact` class should be used providing the appropriate `dwc:measurementID` in the `dwc:Occurrence`. Because the DwC XML schema does not define any constraint on the duplication of ID terms inside an XML element representing a DwC class [97], we can document multiple instances of the `dwc:MeasurementOrFact` class and then refer to them in `dwc:Occurrence` or `dwc:Event` instances using the `dwc:measurementID` term as a link between the records. A guide explaining how to document plant-pollinator interactions using XML is provided along side the vocabulary [98].

Plant-pollinator interactions data as RDF

Representing interactions, or any relations, in DwC using RDF is limited due to the reasons presented before. However, we can still document interactions in RDF according to the data model proposed, if an additional RDF vocabulary is provided. The Darwin-SW (`dsw`, Baskauf and Webb [86]) is a RDF vocabulary designed to complement the Darwin Core Standard and when used in conjunction with Darwin Core IRI terms (`dwciri`) allows to document biological interactions using RDF.

In RDF the interactions are still represented using the `dwc:Event` class, but the link between instances of the `dwc:Occurrence` class and the `dwc:Event` class are made using the `dsw:atEvent` term as predicate in the RDF triplet, having an instance of `dwc:Occurrence` as the subject and an instance of `dwc:Event` as the object (Figure 3). The type and direction of an interaction are given naturally by the RDF triplet composed by two instances of the `dwc:Occurrence` class as subject and object, and a term from the Relations Ontology as predicate (Figure 3).

Additionally, the `dwc:MeasurementOrFact` class should be used to document any other characteristics of the interactions or the occurrences using terms from the plant-pollinator vocabulary to specify the properties `dwciri:measurementType` (for non-literal objects) and `dwc:measurementType` (for literal objects). For terms in the plant-pollinator vocabulary which recommend the adoption of a controlled vocabulary, the term `dwc:measurementValue` can be used with literal objects providing `xsd:string` as the `rdf:datatype` attribute of the `dwc:measurementValue`. Alternatively, a more appropriate solution is to use a non-literal object (URI of the term in the controlled vocabulary) with `xsd:anyURI` as the `rdf:datatype` attribute. Instances of `dwc:Event` and `dwc:Occurrence` classes are then linked to the instances of `dwc:MeasurementOrFact` using the term `dcterms:relation` from Dublin Core (purl.org/dc/terms/), following the Darwin Core RDF Guide [99]. The Plant-Pollinator Interactions vocabulary includes a guide explain how to document plant-pollinator interactions using RDF [100].

Although, we recommend the adoption of Relations Ontology terms as predicates for documenting interactions, terms from other vocabularies and ontologies can be used as well. Similarly, terms from other vocabularies can be used to document any additional measurements or facts about the interactions and occurrences.

Documenting plant-pollinator interaction networks

Plant-pollinator interactions are widely studied using complex interaction networks [101, 102]. Interaction networks are usually documented as adjacency (binary or not) matrices, where nodes represent the species and the edges represent the interactions between nodes. Although, prior to the construction

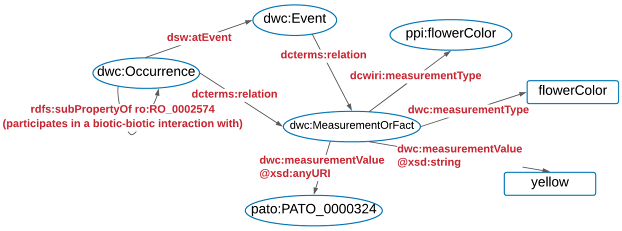

Figure 3. Simplified diagram of a graph structure that can be used to represent biological interactions in conjunction with plant-pollinator interactions vocabulary using RDF. Note that the diagram shows links between instances of classes, but for simplicity only the class URIs of those instances are indicated in the ovals. `dsw:atEvent` is an abbreviation for http://purl.org/dsw/atEvent; `ppi:flowerColor` is an abbreviation for http://rs.rebipp.org.br/ppi/flowerColor; `dcterms:relation` is an abbreviation for http://purl.org/dc/terms/relation; `dwc:` and `dwciri:` are abbreviations for http://rs.tdwg.org/dwc/terms/ and http://rs.tdwg.org/dwc/iri/, respectively.

.

of such networks, the interactions are sampling in field or inferred from pollen grains obtained from the animal's body, most of the plant-pollinator interaction data found in the literature are summarized as interaction networks.

In this particular case, the plant-pollinator schema and vocabulary presented here can also be used to document the interactions at the record-level. The solution is to document each edge (i.e. interaction) in the network as a `dwc:Event` in the proposed schema. The `dwc:Occurrence` does not requires to specify the number of individuals participating on the interaction, and, each node in the network can be documented as a `dwc:Occurrence` of a particular taxon (since the interaction networks have a defined spatial and temporal scopes).

Since the interaction networks have the minimal information needed to represent an interaction in the proposed model, there is no impediment to document them in same way of the other use cases.

Conclusions

By presenting a vocabulary of terms to assist digitization, sharing, aggregation and use of plant-pollinator interactions data, this work provides the means to represent such data in a more complete, accurate and standardized way. This vocabulary can contribute to overcoming some of the technical limitations that have hindered the analyses of large quantities of available data, thus helping to fill an important, long-standing knowledge gap. The process required the involvement and effort of a diverse group of specialists during a period of five years, and has provided a great opportunity to gain experience around community-driven vocabulary building, which we share for future initiatives.

Although this model was conceived for plant-pollinator interactions, we expect that it can be adopted as a general framework for different types of ecological interactions along the antagonism-mutualism continuum, by providing appropriate controlled vocabularies for `dwc:measurementType` and `obis:measurementTypeID`.

The plant-pollinator interactions vocabulary was designed to be fully compatible with the Darwin Core Standard, so that data that uses it can be easily aggregated by current practices of biodiversity data portals. Despite data portals like GBIF do not index all terms contained in datasets (e.g., measurement or facts), and therefore direct searches for some terms are not always possible, the data are still broadly and openly available in a standardized form and can be retrieved from those same portals. To explore and to take full advantage of plant-pollinator standardized data, we are developing an information system and a database of plant-pollinator interactions, as part of the Safeguarding Pollinators and Pollination Services (SURPASS) project [103]. The system will be publicly available on the REBIPP website and function as a data portal of plant-pollinator interactions integrating the biodiversity network for sharing data among member nodes. The system will allow searching for specific interactions records using terms in the plant-pollinator vocabulary for filtering data. The open data policy will guarantee access to plant-pollinators standardized data to many communities around the globe.

This work is also aligned with and part of the Biological Interactions Data Interest Group of the Biodiversity Information Standards [104] organization, which aims to standardize biological interactions data, and we expect the plant-pollinator interactions vocabulary to help achieve this goal. The adoption of a standard depends on how well it can accommodate a wide gamut of use cases without over-compromising its simplicity. Thus, the participation of multiple international actors from the Pollination Biology and Biodiversity Informatics communities, gathered around TDWG was essential for a solution that satisfies, as much as possible, the needs and expectations of most potential stakeholders and helps to advance science in this field. We expect that the plant-pollinator interaction standard and the information system will enable data aggregation from a variety of sources worldwide at higher levels than we have experienced so far, significantly amplifying the plant-pollinator data availability for global synthesis, and contributing to the knowledge base that allows developing responsible ecosystem restoration, biodiversity conservation and sustainable agriculture.

Availability of supporting data and materials

The plant-pollinator interactions vocabulary presented in this article is available in the GitHub repository, https://github.com/rebipp/ppi.

Declarations

List of abbreviations

Ethical Approval (optional)

Not applicable

Consent for publication

Not applicable

Competing Interests

The author(s) declare that they have no competing interests.

Funding

This work was funded by the "São Paulo Research Foundation" (FAPESP) within the project "Safeguarding pollination services in a changing world: theory into practice" (SURPASS2). FAPESP:2018/14994-1. PI: Antonio Mauro Saraiva. The funder had no role in conducting the research and/or during the preparation of the article.

Author's Contributions

Role	Authors
Conceptualization	JAS; PFZ; AMS; KA; EAC; AKV; TCG; JSS
Supervision	AMS; KA; PFZ
Project administration	AMS; KA
Investigation	All authors
Formal Analysis	JAS; PFZ; AMS; DPD; FMS; PJB
Methodology	JAS; AMS; PFZ; DPD; FMS; KA; AKV
Funding Acquisition	AMS; KA
Writing - Original Draft Preparation	JAS; AMS; PFZ; DPD; FMS; KA; MW; IGV; LF; MMM; ARR
Writing - Review & Editing	All authors

Acknowledgements

The authors acknowledge the support of the "Safeguarding pollination services in a changing world: theory into practice (SURPASS2)" project, funded under the Newton Fund Latin America Biodiversity Programme: Biodiversity—Ecosystem Services for Sustainable Development, grants awarded by the Natural Environment Research Council of Great Britain (NERC) [NE/S011870/1], the National Scientific and Technical Research Council of Argentina (CONICET) [RD 1984/19], the São Paulo Research Foundation (FAPESP) [2018/14994-1] and the Chilean Agency of Research and Development (ANID) [NE/S011870/1].

JAS acknowledges the support of Brazilian National Council for Scientific and Technological Development - (CNPq) for a research fellowship (142028/2016-9) and the São Paulo Research Foundation - FAPESP for a research fellowship (2019/27179-7).

AMS acknowledges the support of Brazilian National Council for Scientific and Technological Development - (CNPq) grant number 312.605/2018-8.

ACA thanks the Brazilian National Council for Scientific and Technological Development - (CNPq) for a research fellowship (310999/2018-9).

A.N. thanks the Brazilian National Council for Scientific and Technological Development - (CNPq) (grant 434692/2018-2) and the São Paulo Research Foundation through a Young Investigators Grant - FAPESP (grant number 2019/19544-7).

LGC thanks to Brazilian National Council for Scientific and Technological Development - CNPq (grants: 421668/2018-0; 305157/2018-3) and Fundação para a Ciência e Tecnologia (LISBOA-01-0145-FEDER-028360/EUCLIPO).

The authors acknowledge all participants of the discussions and workshops for the elaboration of the plant-pollinator vocabulary: Silvana Buzato, Alessandra R Pinto, Amanda C Bovo, Ana L Assad, Ana T O Leite, Andrea F P Nunes, Astrid M P Kleinert, Beatriz L Monteiro, Blandina F Viana, Brayan Cavalcante, Bruno L de Faria, Bruno N Silva, Caio S Ballarin, Carlos A Joly, Claúdia I da Silva, Cristiano Menezes, Dora Canhos, Edson Souza, Fabiana R Filardi, Fernando F Jeronimo, João Gonçalves, João Lanna, Joice R M Reis, Karla P T Brancher, Leilane A Bezerra, Liedson T Carneiro, Marina L Mohallem, Marsal D de Amorim, Patricia K Rogeri, Paula Leitman, Paulo E Oliveira, Reisla Oliveira, Sheina Koffler, Vera L Imperatriz-Fonseca, Viviany T do Nascimento and John Wieczorek.

References

1. Potts SG, Imperatriz-Fonseca V, Ngo HT, Aizen MA, Biesmeijer JC, Breeze TD, et al. Safeguarding Pollinators and Their Values to Human Well-Being. Nature 2016 Dec;540(7632):220–229.
2. Ollerton J, Winfree R, Tarrant S. How Many Flowering Plants Are Pollinated by Animals? Oikos 2011;120(3):321–326.
3. Klein AM, Vaissière BE, Cane JH, Steffan-Dewenter I, Cunningham SA, Kremen C, et al. Importance of Pollinators in Changing Landscapes for World Crops. Proceedings of the Royal Society B: Biological Sciences 2007 Feb;274(1608):303–313.
4. Tylianakis JM, Didham RK, Bascompte J, Wardle DA. Global Change and Species Interactions in Terrestrial Ecosystems. Ecology Letters 2008 Dec;11(12):1351–1363.
5. Rodger JG, Bennett JM, Razanajatovo M, Knight TM, van Kleunen M, Ashman TL, et al. Widespread Vulnerability of Flowering Plant Seed Production to Pollinator Declines. Science Advances 2021;7(42):eabd3524.
6. CDB. Report of the Sixth Meeting of the Conference of the Parties to the Convention on Biological Diversity (UNEP/CBD/COP/20/Part 2) Agricultural Biological Diversity Decision VI/5. The Hague; 2002.
7. Schmeller DS, Bridgewater P. The Intergovernmental Platform on Biodiversity and Ecosystem Services (IPBES): Progress and next Steps. Biodiversity and Conservation 2016 May;25(5):801–805.
8. IPBES, Potts SG, Imperatriz-Fonseca VL, Ngo HT, editors, Assessment Report on Pollinators, Pollination and Food Production. Secretariat of the Intergovernmental Science-Policy Platform on Biodiversity and Ecosystem Services; 2016. https://doi.org/10.5281/zenodo.3402857.
9. Dias B, Raw A, Fonseca V. International Pollinators Initiative: The São Paulo Declaration on Pollinators. Report on Th Recommendations of the Workshop on the Conservation and Sustainable Use of Pollinators in Agriculture with Emphasis on Bees. Brazilian Ministry of the Environment (MMA); 1999.
10. FAO's Global Action on Pollination Services for Sustainable Agriculture | Food and Agriculture Organization of the United Nations;. https://www.fao.org/pollination/en/ [Accessed: 24 Jan 2022].
11. Pollinator Conservation | U.S. Geological Survey;. https://www.usgs.gov/centers/eesc/science/pollinator-conservation [Accessed: 24 Jan 2022].
12. EU Pollinators, EU Pollinators - Environment - European Commission;. https://ec.europa.eu/environment/nature/conservation/species/pollinators/index_en.htm [Accessed: 24 Jan 2022].
13. Wolowski M, Ashman TL, Freitas L. Meta-Analysis of Pollen Limitation Reveals the Relevance of Pollination Generalization in the Atlantic Forest of Brazil. PLOS ONE 2014 Feb;9(2):e89498.
14. Menz MHM, Phillips RD, Winfree R, Kremen C, Aizen MA, Johnson SD, et al. Reconnecting Plants and Pollinators: Challenges in the Restoration of Pollination Mutualisms. Trends in Plant Science 2011 Jan;16(1):4–12.
15. Saraiva AM, Imperatriz-Fonseca VL, Cunha RS, Cartolano-Júnior EA. WebBee - a Web-based Information Network on Bees. Revista de Engenharia de Computação e Sistemas Digitais 2003;(1):77–86.
16. Redhead JW, Coombes CF, Dean HJ, Dyer R, Oliver TH, Pocock MJO, et al. Plant-Pollinator Interactions Database for Construction of Potential Networks. NERC Environmental Information Data Centre; 2018.
17. Center for Plant Conservation, Plant-Pollinator Interaction Explorer; 2020. https://plant-pollinator.shinyapps.io/shinyapp/.
18. Poelen JH, Simons JD, Mungall CJ. Global Biotic Interactions: An Open Infrastructure to Share and Analyze

Species-Interaction Datasets. Ecological Informatics 2014 Nov;24:148–159.
19. Simons JD, Yuan M, Carollo C, Vega-Cendejas M, Shirley T, Palomares ML, et al. Building a Fisheries Trophic Interaction Database for Management and Modeling Research in the Gulf of Mexico Large Marine Ecosystem. Bulletin of Marine Science 2013;89(1):135–160.
20. Poisot T, Baiser B, Dunne JA, Kéfi S, Massol F, Mouquet N, et al. Mangal - Making Ecological Network Analysis Simple. Ecography 2015 Jul;p. n/a–n/a.
21. Interaction Web DataBase;. http://www.ecologia.ib.usp.br/iwdb/ [Accessed: 24 Jan 2022].
22. Beas-Luna R, Novak M, Carr MH, Tinker MT, Black A, Caselle JE, et al. An Online Database for Informing Ecological Network Models: http://kelpforest.ucsc.edu. PloS one 2014 Jan;9(10):e109356–e109356.
23. LIFEWEBS PROJECT;. http://www.lifewebs.net/ [Accessed: 24 Jan 2022].
24. Thompson RM, Brose U, Dunne JA, Hall RO, Hladyz S, Kitching RL, et al. Food Webs: Reconciling the Structure and Function of Biodiversity. Trends in Ecology & Evolution 2012 Dec;27(12):689–697.
25. Fortuna MA, Ortega R, Bascompte J. The Web of Life. arXiv:14032575 [q-bio] 2014 Mar;.
26. Hortal J, de Bello F, Diniz-Filho JAF, Lewinsohn TM, Lobo JM, Ladle RJ. Seven Shortfalls That Beset Large-Scale Knowledge of Biodiversity. Annual Review of Ecology, Evolution, and Systematics 2015 Dec;46(1):523–549.
27. Vizentin-Bugoni J, Maruyama PK, de Souza CS, Ollerton J, Rech AR, Sazima M. Plant-Pollinator Networks in the Tropics: A Review. In: Dáttilo W, Rico-Gray V, editors. Ecological Networks in the Tropics: An Integrative Overview of Species Interactions from Some of the Most Species-Rich Habitats on Earth Cham: Springer International Publishing; 2018.p. 73–91.
28. Schemske DW, Mittelbach GG, Cornell HV, Sobel JM, Roy K. Is There a Latitudinal Gradient in the Importance of Biotic Interactions? Annual Review of Ecology, Evolution, and Systematics 2009;40(1):245–269.
29. Arzabe AA, Aguirre LF, Baldelomar MP, Molina-Montenegro MA. Assessing the Geographic Dichotomy Hypothesis with Cacti in South America. Plant Biology 2018;20(2):399–402.
30. Tilman D. Functional Diversity. In: Levin SA, editor. Encyclopedia of Biodiversity New York: Elsevier; 2001.p. 109–120.
31. Emer C, Galetti M, Pizo MA, Jordano P, Verdú M. Defaunation Precipitates the Extinction of Evolutionarily Distinct Interactions in the Anthropocene. Science Advances 2019 Jun;5(6):eaav6699.
32. Mouillot D, Bellwood DR, Baraloto C, Chave J, Galzin R, Harmelin-Vivien M, et al. Rare Species Support Vulnerable Functions in High-Diversity Ecosystems. PLOS Biology 2013 May;11(5):e1001569.
33. Allen-Perkins A, Magrach A, Dainese M, Garibaldi LA, Kleijn D, Rader R, et al. CropPol: A Dynamic, Open and Global Database on Crop Pollination. Ecology 2021;n/a(n/a):e3614.
34. Pimm SL, Lawton JH, Cohen JE. Food Web Patterns and Their Consequences. Nature 1991 Apr;350(6320):669–674.
35. Wilkinson MD, Dumontier M, Aalbersberg IJ, Appleton G, Axton M, Baak A, et al. The FAIR Guiding Principles for Scientific Data Management and Stewardship. Scientific Data 2016 Mar;3(1):160018.
36. Wieczorek J, Bloom D, Guralnick R, Blum S, D??ring M, Giovanni R, et al. Darwin Core: An Evolving Community-Developed Biodiversity Data Standard. PLoS ONE 2012;7(1):e29715–e29715.
37. GBIF: The Global Biodiversity Information Facility, What Is GBIF?; 2022. https://www.gbif.org/what-is-gbif.
38. Access to Biological Collection Data task group, Access to Biological Collection Data (ABCD), Version 2.06. Biodiversity Information Standards (TDWG); 2007.
39. GBIF/TDWG Multimedia Resources Task Group, Audubon Core Multimedia Resources Metadata Schema (S. Baskauf, Review Manager). Biodiversity Information Standards (TDWG); 2013. http://www.tdwg.org/standards/638.
40. Jones M, O'Brien M, Mecum B, Boettiger C, Schildhauer M, Maier M, et al. Ecological Metadata Language version 2.2.0 2019;https://eml.ecoinformatics.org.
41. Droege G, Barker K, Seberg O, Coddington J, Benson E, Berendsohn WG, et al. The Global Genome Biodiversity Network (GGBN) Data Standard Specification. Database 2016 Jan;2016:baw125.
42. Endresen DTF, Knüpffer H. The Darwin Core Extension for Genebanks Opens up New Opportunities for Sharing Genebank Datasets. Biodiversity Informatics 2012 Jul;8.
43. Brenskelle L, Wieczorek J, Davis E, Wallis NJ, Emery K, LeFebvre MJ, et al., A Community-Developed Extension to Darwin Core for Reporting the Chronometric Age of Specimens; 2021.
44. GBIF Registered Extensions;. https://tools.gbif.org/dwca-validator/extensions.do [Accessed: 24 Jan 2022].
45. Lidicker WZ. A Clarification of Interactions in Ecological Systems. BioScience 1979;29(8):475–477.
46. Global Biotic Interactions: Models in Fashion;. https://www.globalbioticinteractions.org/2018/08/16/models-in-fashion/#darwin-core-archive--encyclopedia-of-life-flavor [Accessed: 24 Jan 2022].
47. Ruggiero M, Saraiva AM. A Pollinators Thematic Network for the Americas. In: The Proceedings of TDWG Bratislava, Slovakia; 2007. .
48. TDWG Wiki Archive;. https://github.com/tdwg/wiki-archive/blob/d77f897a52d96f1bd974d5c438790017b8419fac/twiki/data/DarwinCore/InteractionExtension.txt [Accessed: 24 Jan 2022].
49. Saraiva AM, Cartolano Júnior EA, De Giovanni R, Giannini TC, Correa PLP. Exchanging Especimen Interaction Data Using Darwin Core. In: The Proceedings of TDWG Montpellier, France; 2009. .
50. Cartolano Júnior EA. Proposta de um sistema de informação orientado a serviços sobre a biodiversidade de abelhas. text, Universidade de São Paulo; 2009.
51. Saraiva AM, Gemmill-Herren B, Ruggiero M. A Common Schema for Managing Plant-Pollinator Interaction Data. Report on Progress for GEF/UNEP/FAO Project: "Conservations and Management of Pollinators for Sustainable Agriculture, through an Ecosystem Approach"; 2010. p. 33–33.
52. Carvalheiro LG, Saraiva AM, Giannini TC. Establishing Knowledge Management Systems for Ecological Interactions: The Case of Crop Pollinators 2016 Apr;p. 92–112.
53. Tremblay MS, Aubert S, Barnes JD, Saunders TJ, Carson V, Latimer-Cheung AE, et al. Sedentary Behavior Research Network (SBRN) – Terminology Consensus Project Process and Outcome. International Journal of Behavioral Nutrition and Physical Activity 2017 Jun;14(1):75.
54. Zeng ML, Chan LM. Metadata Interoperability and Standardization - A Study of Methodology, Part II: Achieving Interoperability at the Record and Repository Levels. D Lib Mag 2006;12.
55. Duval E, Hodgins W, Sutton SA, Weibel SL. Metadata Principles and Practicalities. D Lib Mag 2002;8.

56. Pomerantz J. Metadata. The MIT Press Essential Knowledge Series, Cambridge, MA, USA: MIT Press; 2015.
57. Issues · rebipp/ppi;. https://github.com/rebipp/ppi [Accessed: 24 Jan 2022].
58. Plant-Pollinator Interactions vocabulary quick reference guide - REBIPP - Plant-Pollinator Interactions Vocabulary;. https://ppi.rebipp.org.br/terms/ [Accessed: 24 Jan 2022].
59. Plant-Pollinator Interactions Controlled Vocabulary List of Terms - REBIPP;. https://ppi.rebipp.org.br/cv/ [Accessed: 24 Jan 2022].
60. Group VMST. Vocabulary Maintenance Standard; 2017.
61. Group VMST. Standards Documentation Standard; 2017.
62. GitHub;. https://github.com [Accessed: 24 Jan 2022].
63. Darwin Core quick reference guide;. https://dwc.tdwg.org/terms/ [Accessed: 24 Jan 2022].
64. Chapman A, Belbin L, Zermoglio P, Wieczorek J, Morris P, Nicholls M, et al. Developing Standards for Improved Data Quality and for Selecting Fit for Use Biodiversity Data. Biodiversity Information Science and Standards 2020 Mar;4:e50889.
65. Cooper L, Meier A, Laporte MA, Elser JL, Mungall C, Sinn BT, et al. The Planteome Database: An Integrated Resource for Reference Ontologies, Plant Genomics and Phenomics. Nucleic Acids Research 2018 Jan;46(D1):D1168–D1180.
66. PATO - the Phenotype And Trait Ontology;. https://github.com/pato-ontology/pato [Accessed: 24 Jan 2022].
67. Hoehndorf R, Alshahrani M, Gkoutos GV, Gosline G, Groom Q, Hamann T, et al. The Flora Phenotype Ontology (FLOPO): Tool for Integrating Morphological Traits and Phenotypes of Vascular Plants. Journal of Biomedical Semantics 2016 Nov;7(1):65.
68. Yoder MJ, Mikó I, Seltmann KC, Bertone MA, Deans AR. A Gross Anatomy Ontology for Hymenoptera. PLOS ONE 2010 Dec;5(12):e15991.
69. Park CA, Bello SM, Smith CL, Hu ZL, Munzenmaier DH, Nigam R, et al. The Vertebrate Trait Ontology: A Controlled Vocabulary for the Annotation of Trait Data across Species. Journal of Biomedical Semantics 2013 Aug;4(1):13.
70. Mungall CJ, Torniai C, Gkoutos GV, Lewis SE, Haendel MA. Uberon, an Integrative Multi-Species Anatomy Ontology. Genome Biology 2012 Jan;13(1):R5.
71. Buttigieg PL, Morrison N, Smith B, Mungall CJ, Lewis SE, the ENVO Consortium. The Environment Ontology: Contextualising Biological and Biomedical Entities. Journal of Biomedical Semantics 2013 Dec;4(1):43.
72. Smith B, Ceusters W, Klagges B, Köhler J, Kumar A, Lomax J, et al. Relations in Biomedical Ontologies. Genome Biology 2005 Apr;6(5):R46.
73. Bronstein JL. Mutualism. Oxford University Press; 2015.
74. Pedruski MT, Fussmann GF, Gonzalez A. Predicting the Outcome of Competition When Fitness Inequality Is Variable. Royal Society Open Science;2(8):150274.
75. Mazancourt CD, Loreau M, Dieckmann U. Understanding Mutualism When There Is Adaptation to the Partner. Journal of Ecology 2005;93(2):305–314.
76. Martin BD, Schwab E. Current Usage of Symbiosis and Associated Terminology. International Journal of Biology 2012 Nov;5(1):p32–p32.
77. Cassidy C, Grange LJ, Garcia C, Bolam SG, Godbold JA. Species Interactions and Environmental Context Affect Intraspecific Behavioural Trait Variation and Ecosystem Function. Proceedings of the Royal Society B: Biological Sciences 2020 Jan;287(1919):20192143.
78. Werner EE. Individual Behavior and Higher-Order Species Interactions. The American Naturalist 1992;140:S5–S32.
79. Brosi BJ. Pollinator Specialization: From the Individual to the Community. New Phytologist 2016 Jun;210(4):1190–1194.
80. Nakazawa T. Species Interaction: Revisiting Its Terminology and Concept. Ecological Research 2020;35(6):1106–1113.
81. Ontobee: OBI - Class: organism;. http://www.ontobee.org/ontology/OBI?iri=http://purl.obolibrary.org/obo/OBI_0100026 [Accessed: 24 Jan 2022].
82. Jordano P. The Biodiversity of Ecological Interactions: Challenges for Recording and Documenting the Web of Life. Biodiversity Information Science and Standards 2021 Sep;5:e75564.
83. Jordano P. Sampling Networks of Ecological Interactions. Functional Ecology 2016;30(12):1883–1893.
84. Cartolano Jr EA, Saraiva AM, Correa PLP, Giannini TC, Giovanni R. Uma Proposta de Esquema de Dados de Relacionamento Entre Espécimes. In: CLEI/Centro Latinoamericano de Estudios en Informática, editor. XXXIII Conferencia Latinoamericana de Informática - CLEI 2007; 2007. p. 1–8.
85. Pando F. How Species Interactions Are Managed in Plinian Core: Status and Questions. Biodiversity Information Science and Standards 2017 Aug;1:e20556.
86. Baskauf SJ, Webb CO. Darwin-SW: Darwin Core-based Terms for Expressing Biodiversity Data as RDF. Semantic Web 2016 Oct;7(6):629–643.
87. New Term - relationshipOfResourceID · Issue #283 · tdwg/dwc;. https://github.com/tdwg/dwc/issues/283 [Accessed: 24 Jan 2022].
88. Vanderweyen A, Fraiture A, Groom Q, Desmet P, Reyserhove L. Catalogue of the Rust Fungi of Belgium 2019;Version 1.2.
89. Scheinberg L. CAS Herpetology (HERP) 2019;Version 33.15.
90. Cheadle Center for Biodiversity and Ecological Restoration. University of California Santa Barbara Invertebrate Zoology Collection. 2021;Occurrence dataset https://doi.org/10.15468/w6hvhv accessed via GBIF.org.
91. Faulwetter S, Markantonatou V, Pavloudi C, Papageorgiou N, Keklikoglou K, Chatzinikolaou E, et al. Polytraits: A Database on Biological Traits of Marine Polychaetes. Biodiversity Data Journal 2014 Jan;(2):e1024.
92. GBIF: Introduction to sampling-event data;. https://www.gbif.org/sampling-event-data [Accessed: 24 Jan 2022].
93. Darwin Core Maintenance Group, List of Darwin Core Terms. Biodiversity Information Standards (TDWG); 2021. http://rs.tdwg.org/dwc/doc/list/2021-07-15.
94. GBIF - Darwin Core Archive Assistant v1.1;. http://tools.gbif.org/dwca-assistant/ [Accessed: 24 Jan 2022].
95. De Pooter D, Appeltans W, Bailly N, Bristol S, Deneudt K, Eliezer M, et al. Toward a New Data Standard for Combined Marine Biological and Environmental Datasets - Expanding OBIS beyond Species Occurrences. Biodiversity Data Journal 2017 Jan;5:e10989–e10989.
96. Plant-Pollinator Interactions Vocabulary text guide - REBIPP;. https://ppi.rebipp.org.br/text/ [Accessed: 24 Jan 2022].
97. Darwin Core XSD Schema;. https://github.com/tdwg/dwc/blob/master/docs/xml/tdwg_dwcterms.xsd [Accessed: 24 Jan 2022].
98. Plant-Pollinator Interactions Vocabulary XML guide - REBIPP;. https://ppi.rebipp.org.br/xml/ [Accessed: 24 Jan 2022].
99. Darwin Core and RDF/OWL Task Groups, Darwin Core RDF Guide. Biodiversity Information Standards (TDWG); 2015.
100. Plant-Pollinator Interactions Vocabulary RDF guide - REBIPP;. https://ppi.rebipp.org.br/rdf/ [Accessed: 24 Jan

2022].
101. Bascompte J. Mutualistic Networks;7(8):429–436.
102. Memmott J. The Structure of a Plant-Pollinator Food Web;2(5):276–280.
103. SURPASS2;. https://bee-surpass.org/ [Accessed: 24 Jan 2022].
104. Biological Interactions Data - TDWG;. https://www.tdwg.org/community/interaction/ [Accessed: 24 Jan 2022].

Supplementary materal 2

Click here to access/download
Supplementary Material
associatedTaxa_supplementary_material.txt

Click here to access/download
Supplementary Material
ppi_terms_versions_supplementary_material.xlsx
