# Supplementary material for: Data standardization of plant–pollinator interactions
Source: Gigascience. 2022 May 26;11:giac043. doi: 10.1093/gigascience/giac043 (PMC9154084; doi:10.1093/gigascience/giac043)
Supplement: giac043_Response_to_Reviewer_Comments_Original_Submission [file giac043_response_to_reviewer_comments_original_submission.pdf]

Author's Response To Reviewer Comments

Close

Reviewer #1:

- "Regarding this implementation I wonder if two key aspects are sufficiently highlighted (eg Fig 1): the interaction (a single interaction) has consequences (outcomes) for BOTH partners. It seems the dwc:Event has a proper descriptor for the animal (dwc:ResourceRelationship class) but not for the plant: the obis:ExtendedMeasurementOrFact extension appears to deal just with characteristics of the plant, flower, nectar produced, etc, and not with the result or consequence of the interaction for the plant. So, I miss a dwc:ResourceRelationship for the plant (e.g., pollen.is.deposited, flower.damaged, nectar.robbed, etc.) as well as an obis:ExtendedMeasurementOrFact for the pollinator (e.g., morphological characteristics, phenology, etc.). I mean, the structure of relationships between boxes in Fig 1 should be symmetrical."
We changed the figure following the suggestion of the reviewer, and now the figure is symmetrical for plant and animal occurrences. We highlighted that the only on dwc:ResoureRelationship must be used since the interactions have a direction (the subject to the object), but any computational system or user is able to recovery the inverse interaction (e.g visitsFlowerOf -> hasFlowersVisitBy), so no additional dwc:ResourceRelationship is needed.

- "Obviously, depending on the amount of data collected or the specific objectives of the samplings, not all this information can be available. Yet the proposed vocabulary is detailed enough to capture most of the details of interest for any plant-pollinator interaction. I miss a brief comment about the minimal set of descriptors that could be considered to provide adequate information for this type of interaction, like a real world "example". That would be of interest to more general readers."
We added a paragraph about the minimum set of terms in order to provide adequate information about the interactions.

- "1. I'd suggest increasing font size in Fig 3 to improve readability."
The font size was increased. Now the Fig 3 is more readable.

Reviewer #2:

- "The authors emphasized that the interaction may be based on individual level. I agree with this. However, data on plant-pollinator interaction is not always collected at individual level. For example, a pollination network is often constructed based on pollen on pollinators. In such studies, it is impossible to know how many plant individuals have been visited by an individual pollinator. Moreover, a pollinator individual could carry pollen grains from multiple plant species. How can this type of data be treated as an interaction in this study?"
We added a brief section about how to document data in interaction networks ("Documenting plant-pollinator interaction networks"). We hope it clarify how networks can be standardized. It should not be different from the individual-level, since in networks the information is only structured in a different format, but essentially it is the same. Nodes can be documented as dwc:Occurrence and edges/links as dwc:ResourceRelationship, having the dwc:Event as link between dwc:Occurrences, dwc:ResourceRelationship and any measurement or facts which might exists.

- "Should pollinators be identified to species level in the data schema? I could not understand whether an interaction event in the data schema allows the interaction that a pollinator (or a plant) is not identified to species level. This is an important point because we can't always identify pollinators to species level. Moreover, we sometimes can't identify pollinator taxa even to family or genus level. For example, many studies on pollination have identified pollinators as not taxa rather than functional

groups (e.g. "midge", "long-tongued bee" or "small beetle"). Does the data scheme allow interaction between a plant species and a pollinator functional group?"
We added a brief paragraph explain that DwC, and so, the schema presented in the manuscripts, does not make any assumption about the taxonomic rank level that should be given in the dwc:scientificName term. So, it is fine to use any level above (or bellow) species rank to document the organisms in the dwc:Occurrence. Theschema presented does not make any changes in the DwC scientificName term definition: "The full scientific name, with authorship and date information if known. When forming part of an Identification, this should be the name in lowest level taxonomic rank that can be determined." (https://dwc.tdwg.org/terms/#dwc:scientificName).

We would like to thank you the reviewers for the valuable comments and suggestions in order to improve the manuscript.

We hope that all the issues pointed by the reviewers have been addressed.

Sincerely,
José A. Salim

Close
