# Supplementary material for: Data standardization of plant–pollinator interactions
Source: Gigascience. 2022 May 26;11:giac043. doi: 10.1093/gigascience/giac043 (PMC9154084; doi:10.1093/gigascience/giac043)
Supplement: giac043_Reviewer_1_Report_Original_Submission — Pedro Jordano -- 3/15/2022 Reviewed [file giac043_reviewer_1_report_original_submission.pdf]

Reviewer Report

Title: Data standardization of plant-pollinator interactions

Version: Original Submission **Date:** 3/15/2022

Reviewer name: Pedro Jordano

Reviewer Comments to Author:

This is a solid analysis and proposal addressing a most interesting issue in biodiversity data sharing: standards are currently not suitable to fully support interaction data recording and sharing. In recent years, especially since 2000, a great amount of information has been gathered by researchers interested in quantifying the diversity of ecological interactions. Hese interactions between species take many forms (e.g., parasitism, seed dispersal by animals, pollination by animals, plant-mycorrhizae, plant-plant facilitation, host-pathogen, etc.). The interactions vary enormously in natural history details, outcomes, etc. so that a proper recording and characterisation aimed at data sharing needs a robust and formal set of descriptors.

The authors do a honest attempt to develop a vocabulary of terms and a data model for sharing plant-pollinator interactions data based on the Darwin Core standard. They introduce 48 new terms targeting several key aspects of plant-pollinator interactions that can be used to capture information from different approaches and scales. It is an innovative study where the authors present a paradigm change to shift the focus from the organisms themselves to the interactions they have.

Technically, the proposed vocabulary is quite exhaustive and complete. The authors provide solutions for data serialization using RDF, XML and DwC-Archives and recommendations of existing controlled vocabularies for some of the terms. The contribution fully supports open access to standardized data on plant-pollinator interactions.

The manuscript provides an ample introduction, including a historical analysis of the development of terms and efforts to quantify plant-pollinator interactions. The ecological background for formalising the vocabulary is solid and well informed, as expected from the recognized expertise of many of the co-authors. This provides a solid background to the technical aspects of the database and protocol design. For example, I fully agree with a basic concept: the individual perspective of the ecological interactions: what we see in nature are interactions (encounters)between individual partners. Then we usually proceed to collapse these inventories to the species level.

From a more technical perspective (despite I'm not a data expert) I agree that the dwc:associatedTaxa, dwc:ResourceRelationship, and the dwc:associatedOccurrence terms are insufficient to capture all the elements that can be recorded for an ecological interaction. In particular I agree with the authors that the Pinion core term dwc:ResourceRelationship can't fully capture any information regarding the outcome of the interactions, and this key to establish the proper type of interaction itself. For example, flower visitation by insects that act as nectar robbers but may eventually, pollinate the flower.

Regarding this implementation I wonder if two key aspects are sufficiently highlighted (eg Fig 1): the interaction (a single interaction) has consequences (outcomes) for BOTH partners. It seems the dwc:Event has a proper descriptor for the animal (dwc:ResourceRelationship class) but not for the plant:

the obis:ExtendedMeasurementOrFact extension appears to deal just with characteristics of the plant, flower, nectar produced, etc, and not with the result or consequence of the interaction for the plant. So, I miss a dwc:ResourceRelationship for the plant (e.g., pollen.is.deposited, flower.damaged, nectar.robbed, etc.) as well as an obis:ExtendedMeasurementOrFact for the pollinator (e.g., morphological characteristics, phenology, etc.). I mean, the structure of relationships between boxes in Fig 1 should be symmetrical.
Obviously, depending on the amount of data collected or the specific objectives of the samplings, not all this information can be available. Yet the proposed vocabulary is detailed enough to capture most of the details of interest for any plant-pollinator interaction. I miss a brief comment about the minimal set of descriptors that could be considered to provide adequate information for this type of interaction, like a real world "example". That would be of interest to more general readers.
Notes:
1. I'd suggest increasing font size in Fig 3 to improve readability.

Methods

Are the methods appropriate to the aims of the study, are they well described, and are necessary controls included? Choose an item.

Conclusions

Are the conclusions adequately supported by the data shown? Choose an item.

Reporting Standards

Does the manuscript adhere to the journal's guidelines on [minimum standards of reporting?] Choose an item.

Choose an item.

Statistics

Are you able to assess all statistics in the manuscript, including the appropriateness of statistical tests used? Choose an item.

Quality of Written English

Please indicate the quality of language in the manuscript: Choose an item.

Declaration of Competing Interests

Please complete a declaration of competing interests, considering the following questions:

- Have you in the past five years received reimbursements, fees, funding, or salary from an organisation that may in any way gain or lose financially from the publication of this manuscript, either now or in the future?
- Do you hold any stocks or shares in an organisation that may in any way gain or lose financially from the publication of this manuscript, either now or in the future?
- Do you hold or are you currently applying for any patents relating to the content of the manuscript?
- Have you received reimbursements, fees, funding, or salary from an organization that holds or has applied for patents relating to the content of the manuscript?
- Do you have any other financial competing interests?
- Do you have any non-financial competing interests in relation to this paper?

If you can answer no to all of the above, write 'I declare that I have no competing interests' below. If your reply is yes to any, please give details below.

I declare that I have no competing interests.

I agree to the open peer review policy of the journal. I understand that my name will be included on my report to the authors and, if the manuscript is accepted for publication, my named report including any attachments I upload will be posted on the website along with the authors' responses. I agree for my report to be made available under an Open Access Creative Commons CC-BY license (http://creativecommons.org/licenses/by/4.0/). I understand that any comments which I do not wish to be included in my named report can be included as confidential comments to the editors, which will not be published.

Choose an item.

To further support our reviewers, we have joined with Publons, where you can gain additional credit to further highlight your hard work (see: https://publons.com/journal/530/gigascience). On publication of this paper, your review will be automatically added to Publons, you can then choose whether or not to claim your Publons credit. I understand this statement.

Yes Choose an item.
