# Supplementary material for: Data standardization of plant–pollinator interactions
Source: Gigascience. 2022 May 26;11:giac043. doi: 10.1093/gigascience/giac043 (PMC9154084; doi:10.1093/gigascience/giac043)
Supplement: giac043_Reviewer_2_Report_Original_Submission — Daichi Funamoto -- 3/24/2022 Reviewed [file giac043_reviewer_2_report_original_submission.pdf]

Reviewer Report

Title: Data standardization of plant-pollinator interactions

Version: Original Submission **Date:** 3/24/2022

Reviewer name: Daichi Funamoto

Reviewer Comments to Author:

General comment
This study provides a new model for sharing data on plant-pollinator interactions. The data model could contribute to future research on pollination biology. This is a well written paper. I have only a few comments.
1. The authors emphasized that the interaction may be based on individual level. I agree with this. However, data on plant-pollinator interaction is not always collected at individual level. For example, a pollination network is often constructed based on pollen on pollinators. In such studies, it is impossible to know how many plant individuals have been visited by an individual pollinator. Moreover, a pollinator individual could carry pollen grains from multiple plant species. How can this type of data be treated as an interaction in this study?
2. Should pollinators be identified to species level in the data schema? I could not understand whether an interaction event in the data schema allows the interaction that a pollinator (or a plant) is not identified to species level. This is an important point because we can't always identify pollinators to species level. Moreover, we sometimes can't identify pollinator taxa even to family or genus level. For example, many studies on pollination have identified pollinators as not taxa rather than functional groups (e.g. "midge", "long-tongued bee" or "small beetle"). Does the data scheme allow interaction between a plant species and a pollinator functional group?

Methods

Are the methods appropriate to the aims of the study, are they well described, and are necessary controls included? Choose an item.

Conclusions

Are the conclusions adequately supported by the data shown? Choose an item.

Reporting Standards

Does the manuscript adhere to the journal's guidelines on minimum standards of reporting? Choose an item.

Choose an item.

Statistics

Are you able to assess all statistics in the manuscript, including the appropriateness of statistical tests used? Choose an item.

Quality of Written English

Please indicate the quality of language in the manuscript: Choose an item.

Declaration of Competing Interests

Please complete a declaration of competing interests, considering the following questions:

- Have you in the past five years received reimbursements, fees, funding, or salary from an organisation that may in any way gain or lose financially from the publication of this manuscript, either now or in the future?
- Do you hold any stocks or shares in an organisation that may in any way gain or lose financially from the publication of this manuscript, either now or in the future?
- Do you hold or are you currently applying for any patents relating to the content of the manuscript?
- Have you received reimbursements, fees, funding, or salary from an organization that holds or has applied for patents relating to the content of the manuscript?
- Do you have any other financial competing interests?
- Do you have any non-financial competing interests in relation to this paper?

If you can answer no to all of the above, write 'I declare that I have no competing interests' below. If your reply is yes to any, please give details below.

I declare that I have no competing interests.

I agree to the open peer review policy of the journal. I understand that my name will be included on my report to the authors and, if the manuscript is accepted for publication, my named report including any attachments I upload will be posted on the website along with the authors' responses. I agree for my report to be made available under an Open Access Creative Commons CC-BY license (http://creativecommons.org/licenses/by/4.0/). I understand that any comments which I do not wish to be included in my named report can be included as confidential comments to the editors, which will not be published.

Choose an item.

To further support our reviewers, we have joined with Publons, where you can gain additional credit to further highlight your hard work (see: https://publons.com/journal/530/gigascience). On publication of this paper, your review will be automatically added to Publons, you can then choose whether or not to claim your Publons credit. I understand this statement.

Yes Choose an item.
